# Heat-Induced Actuator Fibers: Starch-Containing Biopolyamide Composites for Functional Textiles

**DOI:** 10.1021/acsami.3c08774

**Published:** 2023-10-03

**Authors:** Hossein Baniasadi, Zahra Madani, Mithila Mohan, Maija Vaara, Sami Lipponen, Jaana Vapaavuori, Jukka V. Seppälä

**Affiliations:** † Polymer Technology, School of Chemical Engineering, Aalto University, Kemistintie 1, 02150 Espoo, Finland; ‡ Department of Chemistry and Materials Science, School of Chemical Engineering, Aalto University, Kemistintie 1, 02150 Espoo, Finland

**Keywords:** compatibilization, copolyamide, starch, shape memory actuator, heat-responsive smart textile

## Abstract

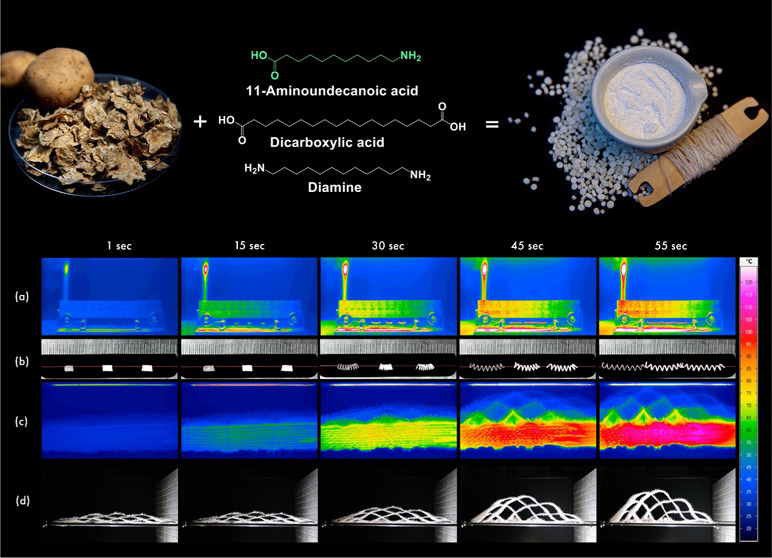

This study introduces the development of a thermally responsive shape-morphing fabric using low-melting-point polyamide shape memory actuators. To facilitate the blending of biomaterials, we report the synthesis and characterization of a biopolyamide with a relatively low melting point. Additionally, we present a straightforward and solvent-free method for the compatibilization of starch particles with the synthesized biopolyamide, aiming to enhance the sustainability of polyamide and customize the actuation temperature. Subsequently, homogeneous dispersion of up to 70 wt % compatibilized starch particles into the matrix is achieved. The resulting composites exhibit excellent mechanical properties comparable to those reported for soft and tough materials, making them well suited for textile integration. Furthermore, cyclic thermomechanical tests were conducted to evaluate the shape memory and shape recovery of both plain polyamide and composites. The results confirmed their remarkable shape recovery properties. To demonstrate the potential application of biocomposites in textiles, a heat-responsive fabric was created using thermoresponsive shape memory polymer actuators composed of a biocomposite containing 50 wt % compatibilized starch. This fabric demonstrates the ability to repeatedly undergo significant heat-induced deformations by opening and closing pores, thereby exposing hidden functionalities through heat stimulation. This innovative approach provides a convenient pathway for designing heat-responsive textiles, adding value to state-of-the-art smart textiles.

## Introduction

Polyamides (PAs) have long been recognized as one of the major engineering thermoplastics, finding applications in various industries such as sports, furniture, textiles, automotive, fishing, and medical devices. Their exceptional properties, including excellent chemical resistance, electrical insulation, robust mechanical and thermal characteristics, as well as ease of processing, have contributed to their widespread use.^1−4^ Recently, PA-based fishing lines have attracted attention for their intriguing application as thermally responsive artificial muscles capable of delivering significant strokes through shape memory effects.^5,6^ These materials offer direct compatibility with textile manufacturing techniques, allowing seamless integration into passive textiles and transforming them into dynamically adaptive three-dimensional (3D) networks.^7^ In this study, we present the design of a predominantly biobased low-melting-point polyamide, which opens up new possibilities in lowering the actuation temperature of previously reported PA-based artificial muscles. Simultaneously, it reduces the reliance of smart textile materials on petroleum-based raw materials. By exploring this approach, we aim to expand the application areas of polyamides and promote sustainable alternatives to the development of smart textiles.

Conventionally, PAs are synthesized from petroleum-based monomers; nevertheless, accumulating more CO_2_ into the atmosphere and environmental concerns emerging from their gradual degradation rates and harmful degradation products, along with the continuous decline in oil reserves, have sparked a growing interest in more environmentally friendly ways of synthesizing PAs.^8,9^ Accordingly, several partially and fully biobased PAs like PA410, PA510, PA1010, and PA11 have been developed recently from natural sources, e.g., castor oil, endowing comparable mechanical strength to traditional petroleum-based PAs like PA6 or PA66.^10,11^ The other well-established approach toward developing more sustainable materials is the incorporation of conventional synthetic polymers, e.g., PAs, with biomass. The production of these biocomposites emits proportionally lower amounts of carbon and greenhouse gases into the atmosphere than plain petroleum-based plastics; furthermore, replacing conventional PAs with these biocomposites can reduce the usage of fossil fuel reserves.^12−15^ Nevertheless, compounding biomass, i.e., biofillers with PAs, is limited by the relatively low thermal stability of biofillers. Moreover, due to the high polarity and rich intermolecular and intramolecular hydrogen bonds in PAs, high processing temperatures, causing decoloring and thermal degradation of biofillers during blending, are needed.^16,17^

Starch is one of the potential carbohydrate candidates for blending with polymers thanks to its abundance, low cost, inherent biodegradability, and renewability.^18,19^ Nevertheless, due to the abundant surface hydroxyl groups, starch suffers from low compatibility with most polymers, e.g., PAs.^20^ Numerous approaches have been developed to enhance starch compatibility with polymer matrices. The most well-known and common method is employing polyethylene-grafted maleic anhydride as a compatibilizer.^21,22^ The grafting of oligomers/polymers is another efficient strategy to enhance interfacial adhesion between starch and polymer matrix.^23,24^ Likewise, disrupting hydrogen bonding between starch molecules via an esterification reaction between hydroxyl groups of starch and succinic anhydrides has also been of interest.^25^ Accordingly, alkenyl succinic anhydrides with different chain lengths, from octenyl succinic anhydride to octadecenyl succinic anhydride, could be used for starch treatment. The size of the alkenyl group and the degree of substitution are important parameters determining the level of starch hydrophobic. Octadecenyl succinic anhydride (OSA) is the most popular alkenyl succinic anhydride, which has been used for a long time for starch hydrophobization. OSA-treated starch containing 3.0 wt % OSA has even been approved by FDA in foods.^26−29^ Besides, it has been used as a sustainable template for biomedical applications.^30^ Although there are many reports on OSA-treated starch as a stabilizer and emulsifier in many food systems,^31,32^ only a few reports are available on its melt blending with polymer matrices.^26,33^

In the current study, OSA was grafted on the surface of starch particles (OSA-*g*-starch) through a simple and solvent-free method to not only strengthen polymer/particle interfacial adhesion but also improve starch dispersion in the matrix significantly. Furthermore, to prevent any thermal degradation of starch particles during compounding, for the first time, a novel biobased polyamide with a remarkably low melting point of 135 °C was synthesized through a copolymerization process. Thanks to the employed compatibilization methods, various concentrations of OSA-*g*-starch, including a very high concentration of 70 wt %, were easily melt-blended with the synthesized copolyamide at 160 °C. To the best of our knowledge, there is no other comprehensive investigation of biopolyamide/starch composites in the literature. Moreover, to showcase the advantages of the prepared biocomposites in shape memory textiles, coiled actuators were prepared and integrated into a woven 3D textile structure. The demonstrator fabric was able to switch between closed and open porous structures as a function of temperature, thus opening the door to the wide variety of multifunctional textiles, where an added functionality could be exposed and hidden on command.

## Experimental Section

### Materials

Starch from potato, 11-aminoundecanoic acid, sodium hypophosphite monohydrate (>99%), trifluoroacetic anhydride (reagentPlus, ≥ 99%), and chloroform-*d* (99.8 atom % D) were obtained from Sigma-Aldrich. Octadecenyl succinic anhydride (mixture of isomers) and 1,12-diaminododecane (≥98%) were prepared from TCI, Japan. Chloroform (for analysis EMPARTA ACS) was purchased from Merck. 1,18-Octadecanedioic acid was bought from Cathay Biotech Company, China.

### Copolymerization

**Figure 1:**
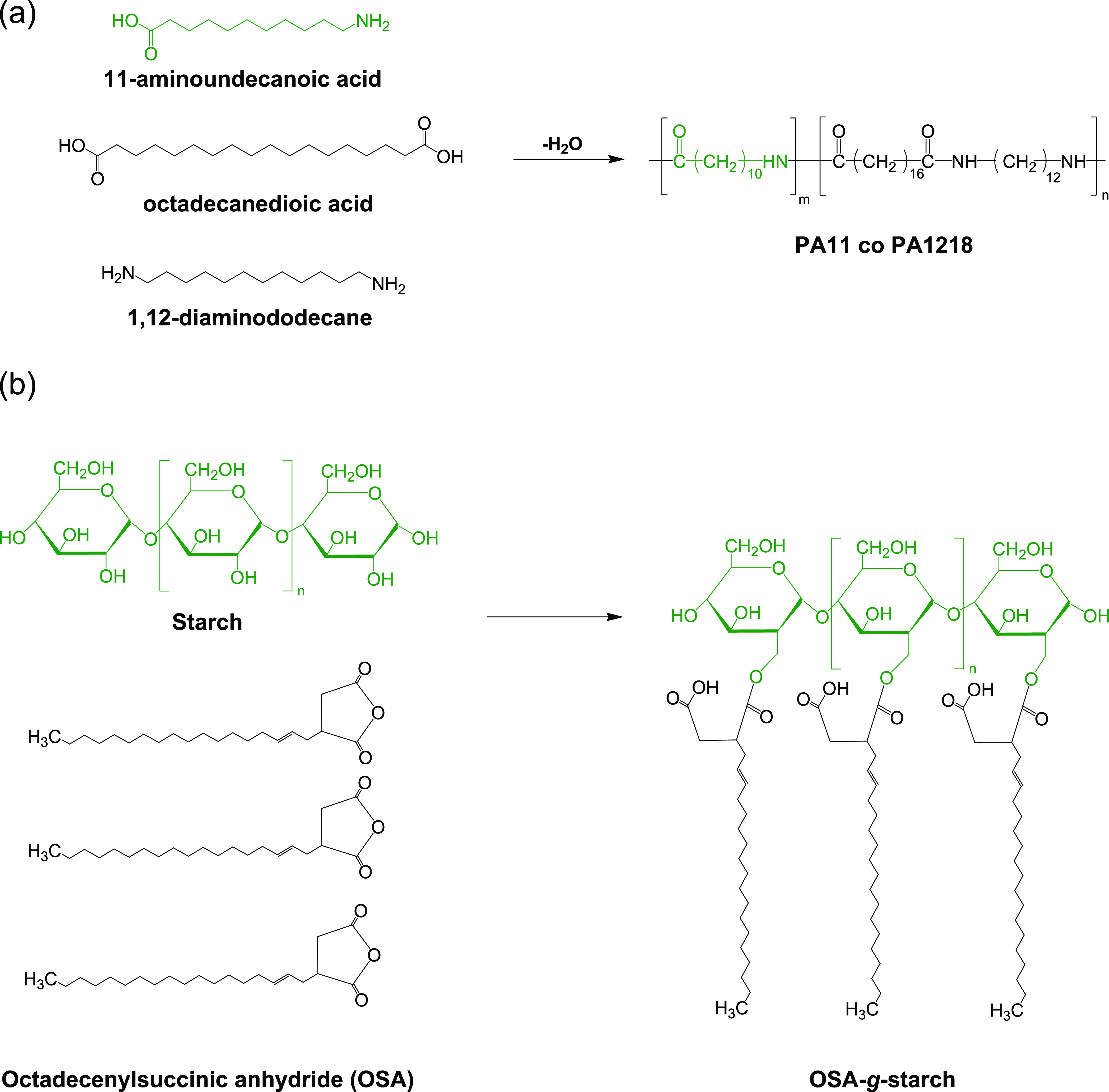
Schematic representation of (a) copolymerization and (b) surface modification of starch with OSA molecules.

The partially biobased polyamide in this study was synthesized through a copolymerization reaction involving two different polyamides, namely, PA11 and PA1218. The reaction process is illustrated schematically in Figure 1a. The monomers, including 11-aminoundecanoic acid, 1,12-diaminododecane, and octadecanedioic acid, were introduced in equal molar quantities into a stainless steel reactor equipped with a heating jacket and an overhead mixer. Sodium hypophosphite monohydrate was added as a catalyst, and the reactor was heated until the temperature reached 200 °C. The monomers were then melted for 1 h under a nitrogen atmosphere at this temperature. Subsequently, the temperature was increased to 240 °C, and the molten monomers/oligomers were gently mixed for 4 h under a nitrogen stream to complete the polycondensation reaction.

After the reaction, the reactor was cooled under a nitrogen flow, and the resulting product, a copolymer of PA11 and PA1218 (termed PA11coPA1218), was collected. The synthesized copolyamide was milled using a Retsch SM 300 Cutting Mill with a 6 mm sieve size (square holes). For comparison, the parent homopolymers, PA11 and PA1218, were also synthesized by using the same polymerization conditions as reference materials.

### Compatibilization

The starch was surface-treated to be compatible with the biopolyamide matrix. The treatment was carried out by grafting octadecenyl succinic anhydride (OSA) via a more eco-friendly approach, i.e., a solvent-free method. Starch was thoroughly dried in a vacuum oven at 70 °C for 48 h. After that, OSA (10 wt % of the starch mass) was mixed gently with the dried starch, and then the mixture was kept in the preheated oven at 100 °C for 24 h. The surface-treated starch was assigned as OSA-*g*-starch and used for blending with the copolyamide matrix. The established reaction between the OSA molecules and starch particles is schematically depicted in Figure 1b. To characterize the surface-treated particles, we dispersed OSA-*g*-starch in toluene at 90 °C and mixed for 1 h. After that, it was filtered and washed with ethanol (Etax B) to remove any unreacted OSA residue.

### Blending and Injection Molding

Different amounts of OSA-*g*-starch were melt-blended with the copolyamide in a counter-rotating twin-screw extruder (Brabender Plasti-Corder PLE 651 with DSK 42/7 twin-screw extruder, The Netherlands) where the screw speed was 20 rpm, and the heating zone temperatures were set to 155 °C (feed), 160 °C (middle), and 160 °C (Die). The output filament was solidified under an air atmosphere and then cut by using a pelletizer. The pellets were then fed to an injection molding machine (Engel ES 200/40) to prepare the tensile test specimens. A gentle dosing procedure was used to avoid any damage to the starch; thereby, the four heating zones were set at 155 °C (feed), 160, 165, and 160 °C (die) while a screw speed of 30% (counter-pressure 1 bar) was used during the dosing step. The injection speed of 150 mm s^–1^ was used during the injection step, followed by after pressure (20 s at 15 bar). The mold temperature was set at 40 °C. For other characterizations, the pellets were hot-pressed for 2 min using a Fontijne Lab Press-TP (The Netherlands) at 160 °C and then cold-pressed at 20 °C for 10 min. The portions of the OSA-*g*-starch in the copolyamide matrix were selected as 10, 30, 50, and 70 wt %, and the samples were coded as PSMS10, PSMS30, PSMS50, and PSMS70, respectively. Biocomposite containing 50 wt % native starch (PNS50) was also prepared to compare its properties with biocomposites containing surface-treated starch.

Plain copolyamide and PASMS10 and PASMS50 composites were chosen for filament production to create actuators. The materials were introduced into a twin-screw microcompounder (Xplore Instruments Midi Extruder). The temperature was set at 160 °C, and the rotational speed was maintained at 15 rpm. The extruded material was solidified on a conveyor operating at a maximum speed of 10 m/min. It should be highlighted that even at a high starch content of 50 wt %, no filament breaking was observed during the extrusion process. The resulting filament was wound around a spindle and utilized for coiled actuator fabrication, as detailed in a subsequent section. The filament diameters for plain copolyamide, PASMS10, and PASMS50 were 190 ± 5, 270 ± 10, and 420 ± 20 μm, respectively.

### Fabrication of Coiled Actuators

PSMS50 was chosen for preparing polymer actuators due to the higher shape fixity (*R*_f_) and shape recovery (*R*_r_), as will be demonstrated in the following section, compared with the other samples. The actuators were fabricated with self-made equipment that resembled the fabrication device. First, the filament twisted until it started to overtwist. The possible maximum number of twists causes a stronger actuation.^34^ After being twisted, the filament was coiled around a metallic mandrel. By varying the twisting and coiling directions, it was possible to make contracting and expanding actuators. An expanding heterochiral structure was used for testing and comparing the actuator properties because expansion was more unlimited as a moving direction. Then, the twisted specimen was thermoset for 150 min at 120 °C in a hydrothermal oven.

### Weaving the Textile Prototype

**Figure 2:**
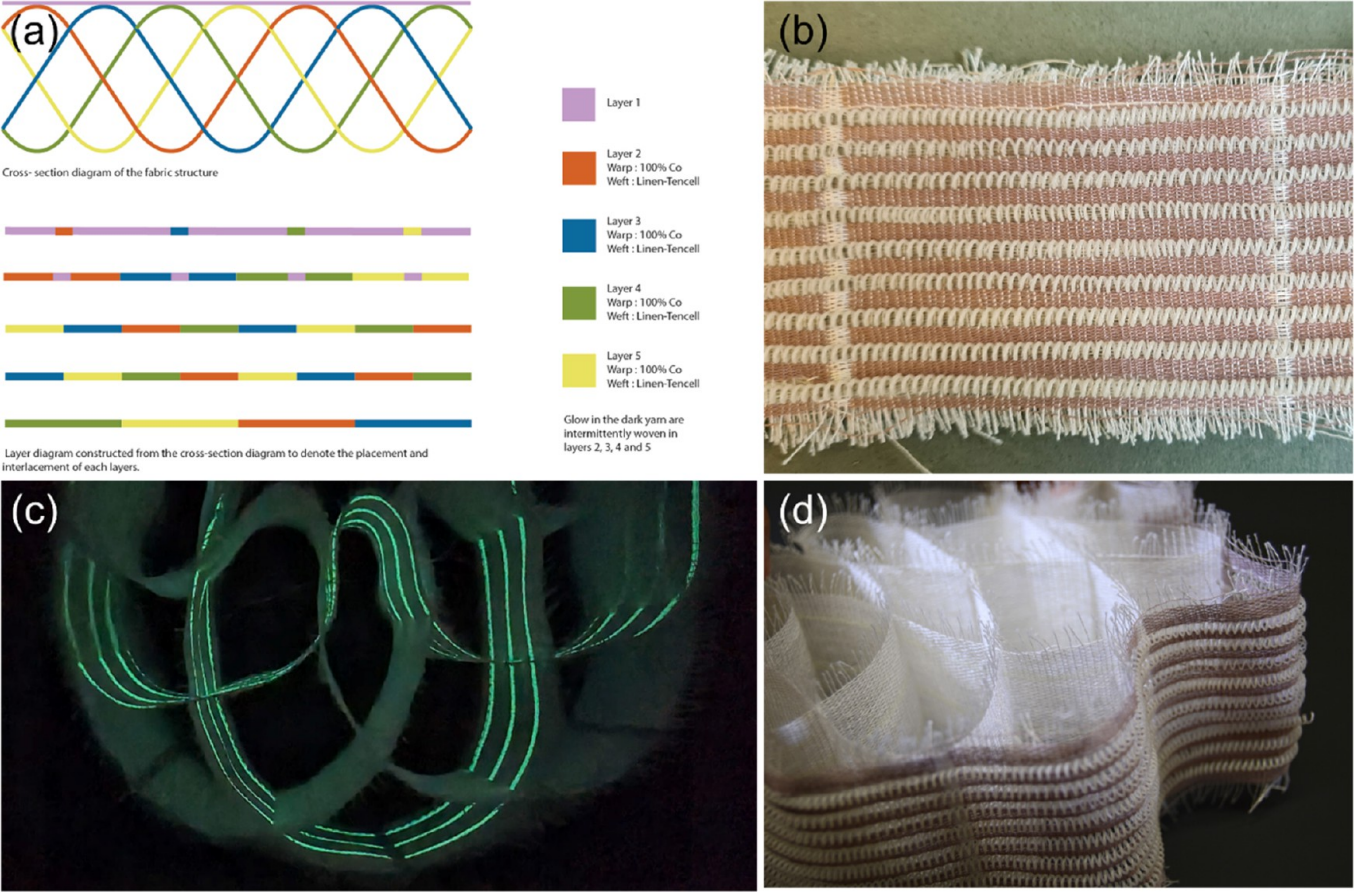
(a) Schematic representation of the developed fabric. The top image shows the cross-sectional diagram, and the bottom image presents the layer diagram constructed from the cross-sectional diagram to denote the placement and interlacement of each layer. In all layers, the warp is 100% cotton. In layer 1, the weft is 100% cotton, and the developed actuators, while in the rest of the layers, the weft is Linen-Tencell. (b) Digital image from the top view of the fabric woven with PSMS50 actuators and cotton in the first layer. (c) Digital image of the fabricated smart textile. (d) Image of fabric photographed in a dark chamber showing the effects of glow-in-the-dark yarn stripes interlacing.

A 3D fabric design was developed by the integration of thermoresponsive PSMS50 coiled actuators into the textile substrate through weaving. The prototype was a multilayered woven fabric that laid as a flat fabric before actuation while on thermal actuation, forming a three-dimensional cellular structure. Figure 2a illustrates the cross-sectional and schematic representation of the layers, while Figure 2b showcases the top view of the fabric woven with PSMS50 actuators and cotton in the first layer. Furthermore, the digital image of the fabricated prototype as well as the image of fabric photographed in a dark chamber are presented in Figure 2c,2d, respectively.

The prototype was woven on a Thread Controller 2 (TC2) Digital Jacquard loom. In a TC2 loom, each warp thread could be programmed to work independently, allowing for the weaving of complex, multilayered fabrics and weave patterns. Moreover, while the warp movements of the loom were computerized, the weft insertion was done manually by hand, further allowing newly developed materials, in this case, PSMS50 coiled actuators, to be prototyped with ease. The five-layered fabric comprised 100% cotton warp and wefts consisting of Linen-Tencel, Cotton, glow-in-the-dark yarn, and PSMS50 actuators. The top layer of the fabric was woven with 100% cotton and PSMS50 actuators, in which the plain weave bonded the actuators to the textile structure so that they did not escape the woven layer during actuation. The cotton yarns and the plain weave structure complemented and aided the actuation of PSMS50 actuators within the textile network. The cotton yarns provided the required flexibility to the top layer, allowing the actuators to contract and expand easily. The weft of the other 4 layers was formed by Linen-Tencel. The linen weft was conducive to creating the diamond structure, as the rigidity of the yarn helped to create well-defined cells that retained their shape without wrinkling during the actuation. This enabled the fabric to open into a fully 3D form during actuation. The interplay of rigid yarns and shrinking yarns in weaving has been tested in earlier work to develop 3D fabrics.^35^ The glow stripes in the dark yarns were intermittently introduced as a supplementary weft in the second and fourth layers. When the fabric was actuated into its three-dimensional form, the glow-in-the-dark yarns peeked through to form glowing interlacing stripes in the dark, as shown in Figure 2c.

### Characterization Methods

Fourier transform infrared spectroscopy (FTIR) was run on a PerkinElmer FTIR with an attenuated total reflection (ATR) machine to investigate the chemical structure of the synthesized PA11, PA1218, and copolyamide as well as starch and OSA-*g*-starch. The spectra were recorded between 4000 and 500 cm^–1^ under a scan rate and resolution of 16 and 4 cm^–1^, respectively. The proton nuclear magnetic resonance (^1^H NMR) spectra conducted by a Bruker AV III 400 NMR spectrometer were used to further evaluate the chemical structure of the homopolymers and copolymer. A mixture of chloroform-*d* and trifluoroacetic anhydride (90/10, V/V) was used to dissolve the sample before subjection to measurement. The gel permeation chromatography (GPC) was performed on an Agilent Multidetector machine to measure the number and average molecular weights, as well as the polydispersity index of the homopolymers and copolymer. A mixture of chloroform (for analysis EMPARTA ACS) and trifluoroacetic anhydride (90/10, V/V) was used as a solvent. Polystyrene standards, dissolved in the same solvent, were used for calibration. The degree of OSA substitution was quantified by an elemental analysis performed on a Thermo Flash Smart CHNSO Elemental Analyzer. First, the calibration curve was plotted considering the measured values of carbon, hydrogen, and nitrogen elements in the native starch. The curve is depicted in Figure S1, where the measured values were graphed versus the theoretical values, that is, C as 44.45%, O as 49.34%, and H as 6.21%. The measured elemental values were then modified due to the calibration curve. After that, the corrected value of the carbon element was employed to calculate the degree of substitution (DS), considering eq 1.
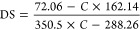
1where 72.06 is the carbon mass in the anhydroglucose unit, *C* is the carbon concentration in the sample (obtained from the elemental analysis), 162.14 stands for the molecular weight of the anhydroglucose unit, and 288.26 and 350.5 are the carbon mass and OSA molecular weight, respectively. The DS was then used to calculate the weight percent of grafted OSA concerning the molecular weight of OSA (350.5 g mol^–1^) and the anhydroglucose unit (162.14 g mol^–1^). The reported values were the mean of three replicates ± the error. Different crystallization properties of the homopolymers and copolymer, including melting point (*T*_m_), crystallization temperature (*T*_c_), melting enthalpy (Δ*H*_m_), crystallization enthalpy (Δ*H*_c_), and degree of crystallinity (χ_c_), were obtained by differential scanning calorimetry (DSC) analysis performed on a TA Instruments MT-DSC Q2000 machine. A two-cycle method was applied under a nitrogen atmosphere, where the thermal history of the sample was removed through the first heating–cooling cycle, and the aforementioned properties were extracted from the second cycle. The temperature range was between −20 and 250 °C, and the heating/cooling scan rate was fixed at 10 °C min^–1^. The crystallinity was calculated by using eq 2, in which Δ*H*^0^ is the enthalpy of a 100% crystalline sample.
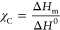
2

For copolyamide, Δ*H*^0^ was calculated considering the weight percent of homopolymers, i.e., PA11 and PA1218, based on eq 3, where 28 and 72% were the weight percents of PA11 and PA1218 in the copolyamide, respectively. The melting enthalpy of a 100% crystalline PA11 was considered as 226.4 J g^–1^,^36^ but since there was no value for a 100% crystalline PA1218 in the literature, the enthalpy of a 100% crystalline PA1212 (292.2 J g^–1^) was used.^37^
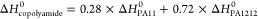
3

DSC was further used to investigate the crystallization properties of the developed composites. A similar thermal cycling test was performed; just the degree of crystallinity was calculated using eq 4, where *x* is the weight percent of filler particles, e.g., OSA-*g*-starch.
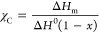
4

The grafting of OSA on starch was qualitatively investigated by thermogravimetric analysis (TGA) thermograms performed on a TA Instruments TGA Q500 machine (Figure S2). Furthermore, the thermal decomposition of the developed biocomposites was monitored by TGA (Figure S3 and Table S1). The sample was heated under a nitrogen atmosphere from room temperature to 800 °C at a heating rate of 10 °C min^–1^. The morphology of the starch particles before and after OSA grafting, as well as the dispersion level of OSA-*g*-starch into the polymer matrix, were evaluated by scanning electron microscopy (SEM) images taken by a Zeiss Sigma VP (Entry-level SEM) machine. The imaging was done from the surface of the particles, while it was carried out from the cryofracture cross-sectional area of plain matrix and biocomposites. A thin layer of gold–palladium was sputtered on the sample surface prior to the subjection of SEM imaging. The mechanical properties of the samples were evaluated by tensile testing done by a Universal Tester Instron 5944 machine. Tensile modulus, yield stress, tensile strength, tensile strain (or elongation at break), and toughness, i.e., the area under the stress–strain curve, were extracted and reported. The test was conducted based on ASTM D638–02, with a stretching rate of 5 mm min^–1^. The samples were conditioned for 48 h at a temperature of 23 °C and a relative humidity of 50%. Each measurement was repeated 5 times, and the mean value ± standard deviation was reported. The thermomechanical characteristics of the samples, including the storage modulus (*E*′), loss modulus (*E*″), and loss factor (tan δ), were evaluated versus temperature by dynamic mechanical analysis (DMA) conducted on a TA Instruments DMA Q800 machine under an air atmosphere. The temperature was ramped up from −20 to 140 °C with a rate of 5 °C min^–1^. The preload, frequency, and strain rate were 1 N, 5 Hz, and 1%, respectively.^37,38^ The glass transition temperature (*T*_g_) was extracted from the tan δ curve.

The shape memory effect of the plain matrix and biocomposites was characterized by Q800 DMA using a controlled-force mode. A strip-shape sample with the dimensions of 5 mm × 3 mm × 0.5 mm (*l* × *w* × *t*) was heated to 80 °C with a heating rate of 5 °C min^–1^ and kept 5 min at this temperature. Then, the force was applied to 18 N at an increasing rate of 0.5 N min^–1^. Subsequently, the sample was cooled to −30 °C followed by a 5 min isothermal step at this temperature. After that, the temporary shape was recovered by removing the load with the rate of −0.5 N min^–1^ until reaching the minimum force of 0.1 N. This process was repeated for 4 cycles. *R*_r_ and *R*_f_ were calculated based on eqs 5 and 6, respectively.^39^
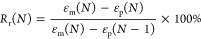
5
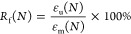
6where ε_m_, ε_p_, ε_u_, and *N* indicate the strain after stretching (before cooling), strain after recovery, strain in the fixed temporary shape, and cycle number, respectively.^39^ It should be highlighted that the upper limit for temperature was set at 80 °C for the plain copolyamide matrix and PSMS10; however, it was 70 °C for PSMS50 because, at higher temperatures, the elongation of the sample was higher than the operating range of the device. The viscosity of the samples and their viscoelastic performance were investigated by melt rheology testing in an oscillatory mode on an Anton Paar Physica MCR 301 machine. The tests were conducted at 160 °C, the same temperature used for melt blending, with parallel geometry (PP25). A strain sweep test was performed from 0.01 to 100% at a fixed angular frequency of 1 Hz to find the linear viscoelastic region. Afterward, the melt strength and flowability were investigated within the linear viscoelastic region, i.e., a fixed strain rate of 1%, through a frequency sweep test ranging from 0.01 to 100 Hz. The trend of both moduli, i.e., storage modulus (*G*′) and loss modulus (*G*″), as well as the complex viscosity (|η*|) versus angular frequency was plotted and explored.

## Results and Discussion

### Copolymerization

**Figure 3:**
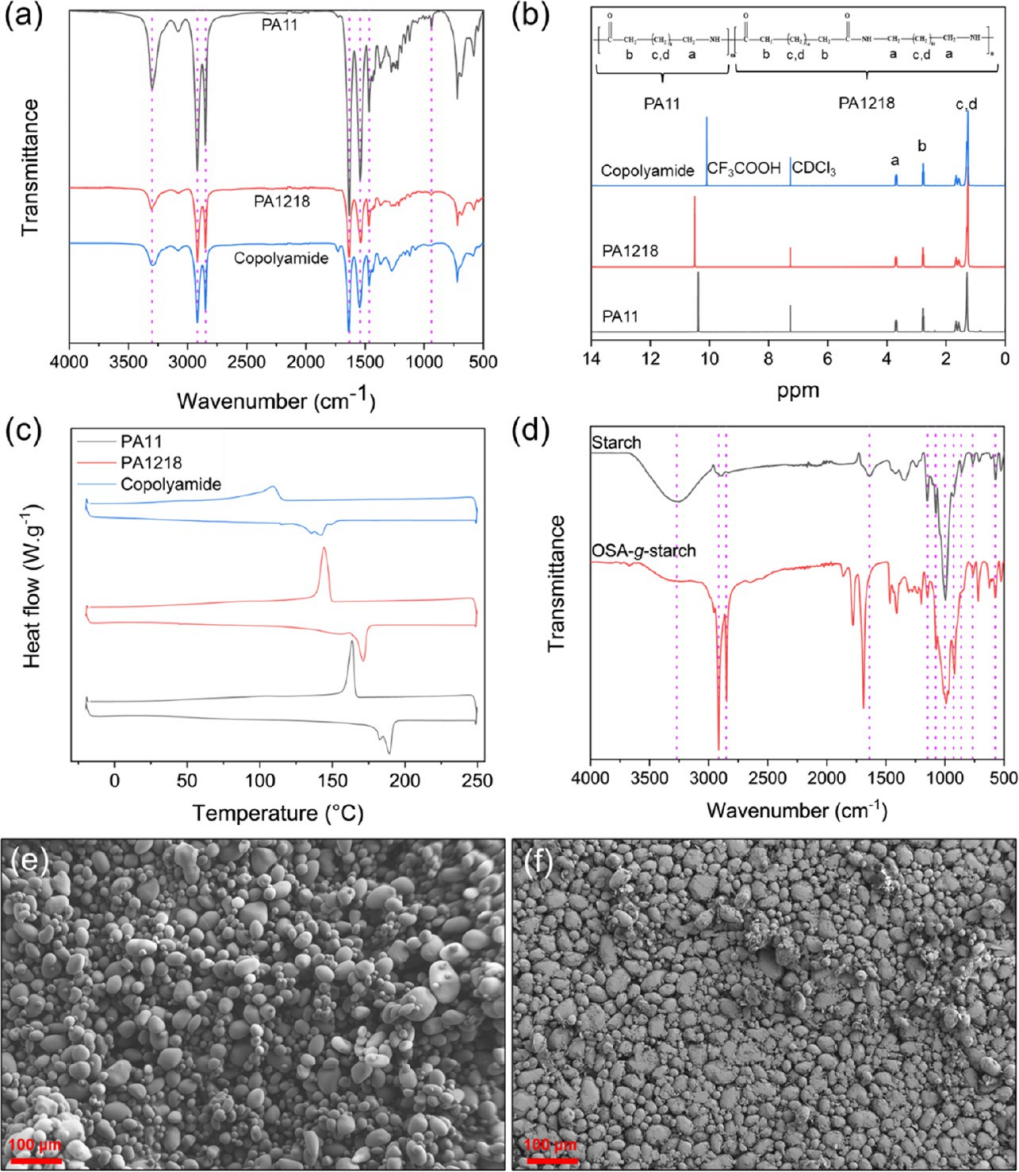
(a) FTIR spectra, (b) ^1^H NMR spectra, and (c) DSC thermograms of the synthesized homopolymers and copolymer. (d) FTIR spectra and (e, f) SEM images of starch before and after treatment with OSA.

FTIR spectroscopy was employed as a fundamental analytical tool to investigate the chemical structures of the homopolymers and copolyamide. As shown in Figure 3a, both homopolymers and copolyamide presented typical polyamides FTIR characteristic peaks. Namely, the N–H group provided a broad bond at 3300 cm^–1^, −CH_2_ asymmetric stretch and symmetric stretch peaks appeared, respectively, at 2917 and 2847 cm^–1^, amide I (C=O) and amide II (−NH–CO−) presented two peaks at 1633 and 1541 cm^–1^, C=O bending formed a bond at 1465 cm^–1^, and a bond at 938 cm^–1^ originated from amide IV.^40,41^ It is noteworthy to mention that the N–H bond peak intensity at 3291 cm^–1^ reduced considerably in PA1218 and copolyamide, indicating a decrease in the amount of amide functional groups, suggesting an increase in the aliphatic segments’ chain length.^11,42,43^

^1^H NMR spectra were used to determine the structure of the synthesized homopolymers and copolyamide concerning the hydrogen-1 nuclei. The results are plotted in Figure 3b. The peak signals at around 10.5 and 7.3 ppm were attributed to the trifluoroacetic anhydride and chloroform-d, respectively. The other resonances that appeared in the range of 1–4 ppm were characteristic peak signals for polyamide^37,38,44^ proving the successful synthesis of the PA11 and PA1218 homopolymers as well as the copolymer through the employed polycondensation reaction. Specifically, the resonance at approximately 3.67 ppm (marked a in Figure 3b) originated from the −CH_2_ proton adjacent to the amino groups, the peak signal at approximately 2.7 ppm (marked b in Figure 3b) was attributed to the −CH_2_ proton adjacent to the carbonyl groups, and the peaks in the range of 1.2 to 1.7 ppm (marked a and d in Figure 3b) were assigned to the −CH_2_ proton in the aliphatic chain.

One of the strengths of the synthesized copolyamide is its relatively low melting point, making it an exciting polymer for compounding with natural fillers, e.g., starch. As such, the melting points of the synthesized homopolymer and copolymer were measured by DSC. The DSC scans are illustrated in Figure 3c. Furthermore, the relevant DSC data extracted from the curves are summarized in Table 1. All polymers showed double melting peaks, a dominant peak accompanied by a minor adjacent shoulder, indicating a polymorphic structure. The appearance of double melting peaks might suggest the melting of different crystalline phases, i.e., α- and γ-forms, or the fusion of the same crystal phase, α- form, yet with varying thicknesses.^10,45,46^ Furthermore, all samples exhibited an exothermic peak corresponding to their crystallization temperature, indicating the formation of a single crystalline phase, even in the copolymer. Noticeably, copolyamide had its own crystallization temperature, not those that appeared for its constituents, indicating that the monomers were not polymerized separately.^47^ This was further supported by GPC results, Table 1 and Figure S4, where the copolyamide had a unimodal molecular weight distribution rather than a bimodal one.

**Table 1:** DSC Data and Different Molecular Weights of the Synthesized Homopolymers and Copolyamide

sample	*T*_c_ (°C)	Δ*H*_c_ (J g^–1^)	*T*_m_ (°C)	Δ*H*_m_ (J g^–1^)	χ_C_ (%)	*M_n_* (g mol^–1^)	*M*_w_ (g mol^–1^)	PDI
PA11	163	41.20	183, 190	41.92	18.20	53,700	91,500	1.70
PA1218	144	52.41	165, 171	32.25	11.04	87,900	15,800	1.79
copolyamide	110	36.42	135, 142	29.70	10.85^a^	72,000	18,600	2.60

a The melting enthalpy of 100% crystalline copolyamide was considered 273.77 J g^–1^ due to eq 3.

On the other hand, copolymer melted and crystallized at relatively lower temperatures than its parent polymers. In addition, its melting and crystallization enthalpies were lower than those of the homopolymers. These results suggest that the copolymerization of different monomers/polymers affected the chain organization, changed the polymer chains’ regularity, and inhibited crystallization in the copolymer, which resulted in lower crystallinity and melting point.^48−50^

### Starch Surface Modification

FTIR spectra were used to investigate the possible covalent bonding between starch and OSA molecules. As plotted in Figure 3d, starch and OSA-*g*-starch exhibited some common peaks, which were characteristic of starch.^26,51^ Specifically, a broad band at 3270 cm^–1^ originated from the intra- and intermolecular hydrogen bonds of O–H stretching, the peaks between 3000 to 2800 cm^–1^ resulted from −CH stretching bonds, a peak at 1643 cm^–1^ assigned to the water molecules trapped in the noncrystalline region of the sample, the peaks located at 927, 1010, 1079, and 1148 cm^–1^ attributed to the −C–O–C– bonds of the anhydroglucose unit, and the bands at 574, 769, and 863 cm^–1^ arose from the C–C stretching and C–H bending vibrations of the glucosidic ring.

The relative intensities of some peaks changed in the OSA-*g*-starch samples. In addition, some new bands appeared, indicating the grafting of the OSA molecules on starch via a covalent reaction between the hydroxyl group of starch and anhydride rings of OSA. For instance, the grafted alkyl chain gave rise to two new peaks at around 2916 and 2850 cm^–1^ in the surface-treated starch. Besides, the relative peak intensity corresponding to hydroxyl groups at around 3300 cm^–1^ decreased significantly, suggesting a lower concentration of hydroxyl groups in the surface-treated sample attributed to their interaction with OSA molecules via esterification. Notably, a newly formed band at 1740 cm^–1^, which could be considered the characteristic of the carbonyl groups in the ester bonds (C=O),^26,38,52^ further proved the claim of the esterification reaction. When the spectra are interpreted, it should be taken into account that the C–C–O stretching and O–C–C stretching bonds of the ester groups at 1240 and 1047 cm^–1^ possibly overlapped with the characteristic peaks of the anhydroglucose unit.

The degree of substitution and the weight percent of the grafted OSA, were estimated based on the elemental analysis. The obtained data are summarized in Table 2. The DS was approximately 5.45 and 2.34 per 100 anhydroglucose units, corresponding to approximately 9.94 and 4.26 wt % OSA in the surface-treated starch before and after washing, respectively. The OSA concentration before washing the sample had an excellent agreement with the added value (10%), as previously explained in the Experimental Section. Approximately half of the added OSA was washed away during the washing process, indicating that the remaining OSA reacted covalently with starch molecules, as observed in the FTIR spectra.

**Table 2:** Elemental Analysis Results of Starch and OSA-*g*-Starch

sample	carbon (%)	hydrogen (%)	nitrogen (%)	oxygen (%)	sulfur (%)	DS^a^	OSA (wt %)
Theoretical Values Regarding the Anhydroglucose Formula, C_6_H_10_O_5_							
starch	44.64	6.19	0	49.16	0		
Measured Values							
Starch	41.33 ± 0.34	6.18 ± 0.09	0	47.55 ± 0.69	0		
OSA-*g*-starch^b^	45.94 ± 0.81	6.92 ± 0.08	0	45.53 ± 0.38	0		
OSA-*g*-starch^c^	43.89 ± 0.09	6.58 ± 0.02	0	47.27 ± 0.08	0		
Corrected Values^d^							
Starch	39.91	6.32	0	46.79	0		
OSA-*g*-starch^b^	48.43	7.10	0	48.00	0	5.45	9.94
OSA-*g*-starch^c^	46.26	6.74	0	49.83	0	2.34	4.26

a Calculated from eq 1.

b Before washing.

c After washing.

d Regarding the calibration curve (Figure S1).

The effect of surface treatment on the morphology of starch particles was monitored using SEM imaging. The micrographs are shown in Figure 3e,3f. The starch granules had an oval geometry with an average diameter of 35 ± 15 μm, in line with the value reported in the literature for different starch types.^53^ Besides, their surface was smooth. After surface treatment, the particle size did not change considerably; however, the surface was no longer smooth and became rough, suggesting a successful grafting of OSA molecules.^54−56^

### Biocomposites Morphology

**Figure 4:**
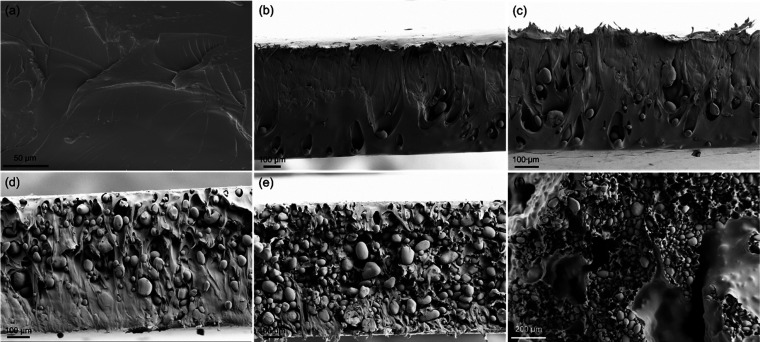
SEM images from cryofracture surface area of (a) neat copolyamide, (b) PSMS10, (c) PSMS30, (d) PSMS50, (e) PSMS70, and (f) PNS50 with 500× magnification.

The microstructure of the biocomposites was evaluated by using cryofracture cross-sectional SEM imaging. The SEM micrographs of the biocomposites, as well as the plain copolyamide and the biocomposite containing 50 wt % of unmodified starch, i.e., PNS50, are shown in Figure 4. The unmodified copolyamide surface was smooth without any cracks and particles, indicating a plain polymer with a tough continuous surface rather than a brittle one. Likewise, no significant surface defects, i.e., pores or cracks, were detected in biocomposites containing surface-treated starch, while OSA-*g*-starch distributed uniformly into the polyamide matrix at a filler content up to 50 wt % with no sign of particle agglomeration. These observations indicated good miscibility of copolyamide and surface-treated starch and improved compatibility and interfacial adhesion between phases,^18,57−59^ achieved via the grafting of OSA on the starch surface.

In contrast, the PNS50 composite containing 50 wt % unmodified starch exhibited significant surface defects along with the formation of voids, confirming that those two substances were inherently incompatible with poor interfacial adhesion between the phases.^60,61^ As can be observed in Figure 4e, the starch reversed from the dispersed phase to the continuous one in PSMS70 with 70 wt % of OSA-*g*-starch, which counts as another piece of evidence of the excellent miscibility of the two phases.^18^

Overall, the average diameter of the starch granules was about 30 μm, which is in good agreement with what was observed previously for the plain OSA-*g*-starch. This indicates that the starch particles were not destroyed under the applied shear stress in the extruder, which has already been introduced in the literature as another reason for the absence of any cracks and folds on the cross-sectional area of the biocomposites.^62^ It is worth notifying that some researchers observed significant agglomerates and defects in the composites with significantly lower starch loadings,^53,63,64^ highlighting the advantage of the employed method for the surface treatment of starch. In this study, successfully incorporating such a high loading of starch particles in the polyamide matrix introduces an eco-friendly, green, durable, and sustainable plastic that may significantly reduce the carbon footprint and greenhouse gas emissions.^65,66^

### Crystallinity Study

The impact of OSA-*g*-starch particles on the crystallization behavior of the developed composites was thoroughly investigated by using DSC. The DSC curves are illustrated in Figure S5, and the corresponding thermal transition data are summarized in Table 3. Interestingly, the composites with up to 30 wt % surface-modified starch particles exhibited double melting peaks akin to those observed in the copolymer. This suggests that the crystal structure remained relatively unchanged at these concentrations. However, at higher starch contents, only a single melting peak was detected. This intriguing phenomenon indicates a transformation from a less ordered γ-crystalline form to a more highly ordered and densely packed α-crystalline structure.^67^ Alternatively, it suggests the formation of the α-crystalline form with a relatively uniform thickness.

**Table 3:** DSC Data of the Synthesized Copolyamide and the Developed Composites

sample	*T*_c_ (°C)	Δ*H*_c_ (J g^–1^)	*T*_m_ (°C)	Δ*H*_m_ (J g^–1^)	χ_C_^a^ (%)
copolyamide	110	36.4	135, 142	29.7	10.9
PSMS10	111	26.8	135, 142	28.3	11.5
PSMS30	115	22.9	135, 143	22.7	11.9
PSMS50	119	18.1	149	17.8	13.0
PSMS70	115	12.4	146	10.0	12.3

a The melting enthalpy of 100% crystalline copolyamide was considered 273.77 J g^–1^ due to eq 3.

Furthermore, the presence of high concentrations of starch led to an increase in the melting point. This can be attributed to the strong hydrogen bonding between starch particles and polymer chains, which restricted the chain mobility and delayed the melting of the polymer chains. The crystallization temperature also shifted to higher values, indicating that the starch particles acted as nucleation sites during polymer crystallization from the melt, lowering the energy barrier of nucleation. As a result, less supercooling of the melt was required to initiate crystallization, leading to a shift in the crystallization temperature to higher values.^68^ Previous studies have reported that higher crystallization temperatures can lead to increased crystallinity in the polymer matrix.^37^ Consequently, the degree of crystallinity (χ_c_) increased from 10.85% in the plain copolymer to 12.96% in the PSMS50 composite. This enhancement confirms that the granular dispersed morphology favored the crystallization of copolyamide and that the nucleation effect was reinforced by the presence of surface-modified starch particles.^22,69^

Moreover, the higher melting point observed in composites with filler contents higher than those of the plain matrix can also be attributed to their relatively higher crystallinity or change in the crystalline phase. It is worth noting that the relatively higher crystallinity in the composites is advantageous, as it can lead to a higher stiffness and tensile modulus. This is because crystalline regions provide stronger and more efficient load transfer pathways.^70^ Additionally, crystalline regions play a significant role in enabling and controlling the shape memory effect, which could prove beneficial in these materials.^71^ These claims and findings will be further investigated in the subsequent sections to gain a deeper understanding of the overall composite behavior and its potential application.

### Mechanical Properties

**Figure 5:**
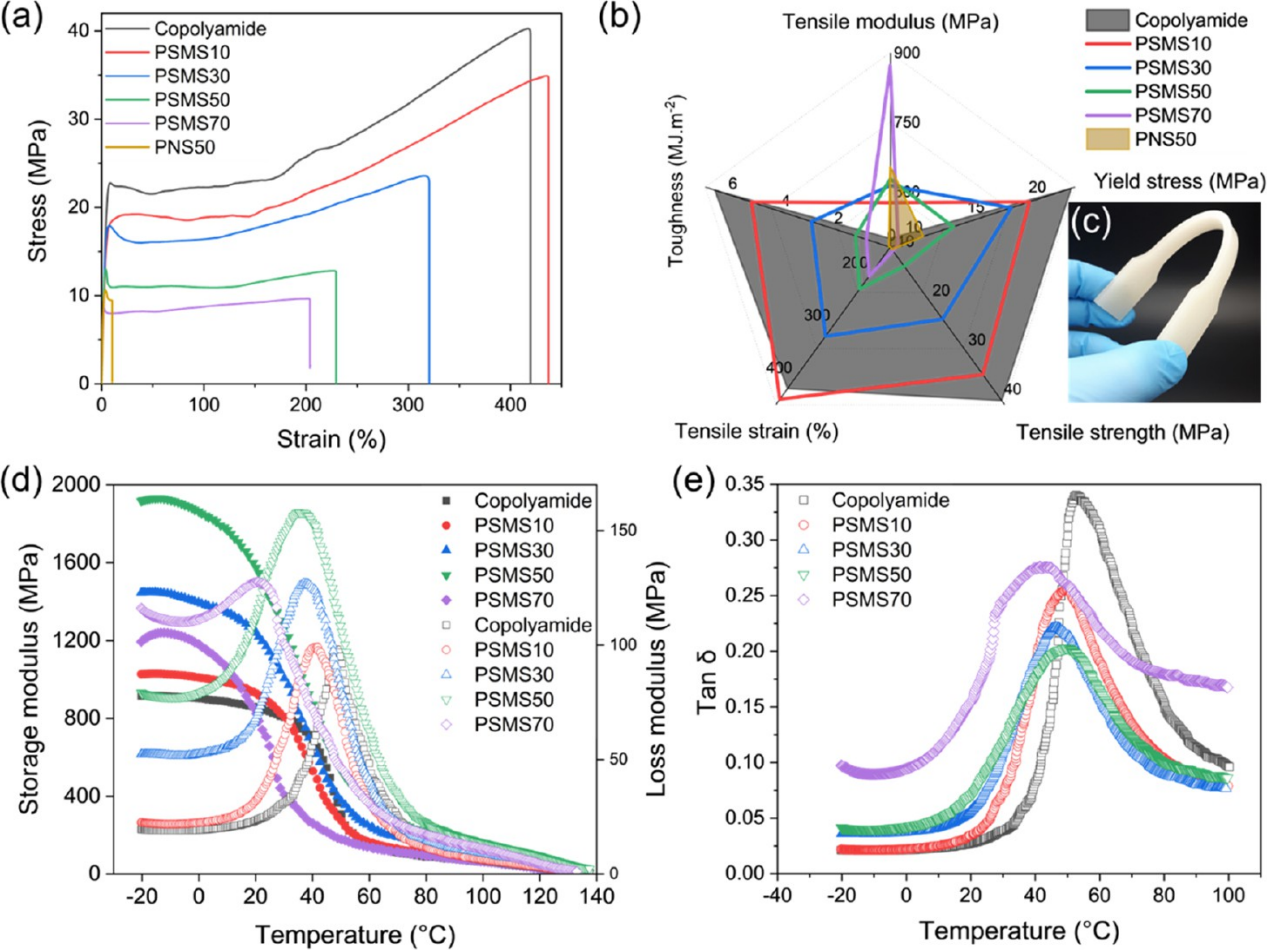
(a) Typical stress–strain curves, (b) comparison of different mechanical properties of the synthesized copolyamide and biocomposites, (c) photograph of bent PSMS70 biocomposite, (d) storage (*E*′) and loss (*E*″) moduli, and (e) loss factor (tan δ) of the synthesized copolyamide and biocomposites versus temperature. The solid and blank symbols represent the storage modulus and loss modulus, respectively.

The mechanical properties are important for a material to be applied, among other things, in the fabrication of textiles. Therefore, different mechanical characteristics of the synthesized copolyamide and developed biocomposites, including the tensile modulus, yield stress, tensile strength, tensile strain, and toughness, were evaluated. The typical stress–strain curves are depicted in Figure 5a. Furthermore, the aforementioned properties are compared in Figure 5b and Table 4. The mechanical properties of the PNS50 biocomposites are also provided for comparison. The plain copolyamide showed a very high elongation of about 420 ± 20%, with tensile modulus and tensile strength of 499 ± 24 and 40.3 ± 1.9 MPa, respectively. While the tensile strength of the copolyamide was in the range reported for commercial PA11^72,73^ and long-chain aliphatic polyamides^37,42,43^ the tensile modulus was significantly lower. In other words, the copolyamide behaved more like a strong and soft polymer,^74^ which could be an advantage for blending with mostly rigid biobased fillers like starch.^75,76^ Accordingly, the yield stress, tensile strength, tensile strain, and toughness were systematically reduced in the biocomposites, while the tensile modulus increased. For instance, in the biocomposite containing 50% surface-modified starch, i.e., PSMS50, tensile strength and tensile strain decreased, respectively, by ∼68 and ∼45%, probably due to the brittleness, rigidity, and poor mechanical properties of starch compared to polyamide. At the same time, the tensile modulus was enhanced by approximately 25%, a notable improvement that can be attributed to the higher crystallinity observed in the composites compared to that of the plain matrix. Crystalline regions within a polymer matrix play a pivotal role in reinforcing mechanical properties, particularly by increasing the material’s stiffness. The alignment and close packing of polymer chains in these crystalline regions facilitate superior load transfer and bolster resistance to deformation, ultimately culminating in enhanced stiffness.^77^ It is worth notifying that the tensile strain dramatically dropped to 10% in the biocomposite containing 50 wt % native starch, i.e., PNS50, proposing a brittle composite. In contrast, it was more than 200% in PSMS70 with an even higher surface-treated filler loading. The photograph captured from bent PSMS70 (Figure 5c) clearly shows the flexibility of this sample. Altogether, the mechanical properties establish the benefit of the employed surface modification method for making starch particles compatible with the polyamide matrix.

**Table 4:** Mechanical Properties of the Synthesized Copolyamide and the Developed Biocomposites

sample	tensile modulus (MPa)	yield stress (MPa)	tensile strength (MPa)	tensile strain (%)	toughness (MJ cm^–3^)
copolyamide	499 ± 24	22.7 ± 1.1	40.3 ± 1.9	420 ± 20	6.7 ± 0.3
PSMS10	576 ± 21	19.3 ± 0.7	34.9 ± 1.3	440 ± 15	5.3 ± 0.2
PSMS30	613 ± 26	17.8 ± 0.7	23.7 ± 1.0	320 ± 14	3.0 ± 0.1
PSMS50	627 ± 35	13.1 ± 0.8	12.9 ± 0.7	230 ± 13	1.3 ± 0.01
PSMS70	872 ± 28	8.6 ± 0.2	9.8 ± 0.3	205 ± 7	0.9 ± 0.01
PNS50	654 ± 38	10.7 ± 0.6	9.5 ± 0.5	10 ± 0.6	0.05 ± 0.01

The trend of copolyamide’s mechanical properties as a function of starch concentration was compared with the research works performed on starch-based thermoplastic composites. A few researchers claimed improvement in the mechanical properties of the polymer matrix after melt blending with starch. For instance, Yusoff et al.^60^ prepared PLA/thermoplastic starch with different compositions and reported around 35% improvement in both tensile modulus and tensile strength of biocomposite with 30% starch, attributed to the good matrix–filler interaction. Nevertheless, similar to our findings, most of the previous studies revealed a significant reduction in the mechanical performance of the matrix. Namely, Landreau et al.^78^ observed an approximately 70% reduction in the tensile strength of the PA11 matrix containing 50 wt % starch content compatibilized by sodium carboxymethylcellulose. Furthermore, Noivoil et al.^23^ used oligo(lactic acid)-grafted starch as a compatibilizer and prepared a PLA/thermoplastic starch biocomposite blend (50/50) with different concentrations of compatibilizer. They reported a 50% reduction in both tensile strength and tensile modulus of the biocomposite but a 9% increase in tensile strain. Similarly, a significant decrease in Young’s modulus and tensile strength of PLA has been reported after blending the matrix with thermoplastic starch.^79,80^ Likewise, Zhang et al.^81^ prepared PLA/starch composites (55/45) compatibilized by maleic anhydride and observed a 15 and 10% reduction in tensile strength and tensile strain, even in the biocomposite containing 2% maleic anhydride as a compatibilizer. Moreover, Zhou et al.^63^ observed a 13% decrease in the tensile strength of PVA/corn starch (80/20) biocomposite.

### Thermomechanical Performance

To further evaluate the OSA-*g*-starch dispersion into the copolyamide matrix, the trends of dynamic storage modulus (*E*′) and dynamic loss modulus (*E*″), as well as the damping factor (tan δ), were evaluated as a function of temperature from −20 to 140 °C by dynamic mechanical analysis (DMA). The results are presented in Figure 5d,5e. Although the moduli were constant at low temperatures, they experienced a sharp reduction of about an order of magnitude between 40 to 60 °C, associated with a peak on tan δ curves, indicating a transition from a glassy state to a rubbery one, also known as glass transition temperature (*T*_g_). This sharp reduction of moduli around *T*_g_ can be explained by the fact that at low temperatures, the molecular segmental motions were frozen and thereby caused a high level of *E*′ from 900 to 1900 MPa. Upon increasing the temperature, the rigid segmental structure relaxed gently, resulting in higher molecular chain motions in the system.^82^ On the other hand, below *T*_g_, both *E*′ and *E*″ increased systematically with the increase in the OSA-*g*-starch content, which could be due to the hydrogen bonds formed between the hydroxy group of starch and amine groups of polyamide. It should be highlighted that the modulus at high temperatures showed a slight improvement after the matrix was blended with surface-modified starch particles, particularly in the PSMS50 composite. This enhancement could be highly advantageous for shape memory applications. The modulus of a material refers to its stiffness or resistance to deformation. In the case of shape memory polymers (SMPs), the modulus at high temperatures plays a critical role in determining the recovery stress and, consequently, the overall performance of SMPs in various applications.^83−85^ When an SMP is deformed above its switching temperature (*T*_g_ or *T*_m_)—in this specific instance, *T*_g_—the high-temperature modulus governs the force it can generate during shape recovery. Achieving a balance between the modulus at high and low temperatures is essential for efficient shape memory behavior. Moreover, the incorporation of surface-modified starch particles in the PSMS50 composite has demonstrated the potential for enhancing the high-temperature modulus, further contributing to improved shape memory properties, as will be discussed in the following sections.

As the temperature increased, the flexible long-chain structure of grafted OSA molecules reduced the rigidity of biocomposites.^86^ Likewise, the peak in tan δ curves, *T*_g_, shifted to lower temperatures upon increasing OSA-*g*-starch content. Namely, *T*_g_ shifted from 54 °C in the copolyamide matrix to 48 °C in PSMA50 and then to 40 °C in PSMS70. This phenomenon suggests that the biocomposites became less viscous after the temperature was raised, and more molecular chain mobility was achieved. In other words, the flexibility of the matrix was developed due to the uniform dispersion of filler particles, which acted mainly as plasticizers rather than reinforcing agents.^87,88^ The reduction in *T*_g_ upon introducing starch particles might be an advantage for thermoresponsive shape memory polymer actuators because the actuators could start to activate at lower temperatures.

A single *T*_g_ appears in a fully miscible polymer composite system, lying between the *T*_g_ of the individual polymers.^58,89^ The *T*_g_ for potato starch with 13–15% moisture content is reported to be between 75 and 95 °C.^90^ Accordingly, a well-defined peak in the loss factor indicated effective miscibility between polyamide and starch as well as a homogeneous blend microstructure. On the other hand, the height of the tan δ curve pertains to the matrix/filler interphase internal energy dissipation.^87^ The height of tan δ was relatively lower in biocomposites than in the plain matrix, indicating that the addition of OSA-*g*-starch particles considerably reduced the viscoelastic damping factor of the polyamide matrix, and accordingly, the sample became less rigid.

### Rheology and Processability

**Figure 6:**
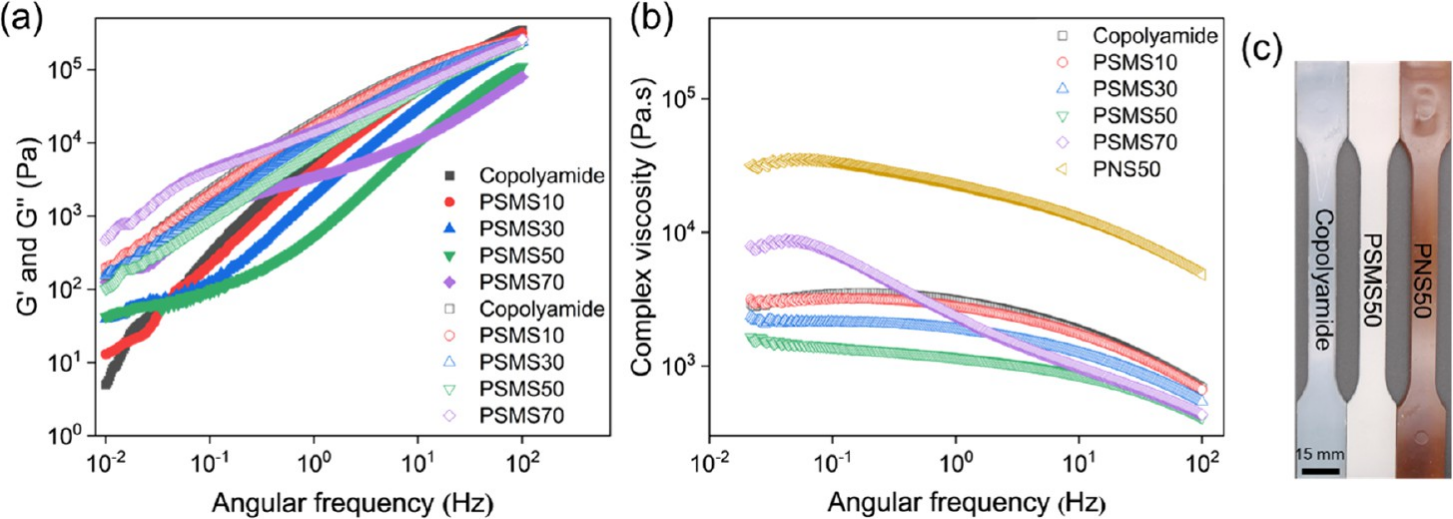
(a) Storage (*G*′) and lost (*G*″) moduli and (b) complex viscosity (|η*|) of the synthesized copolyamide and biocomposites versus angular frequency at a fixed strain rate of 1% and temperature of 160 °C. The solid and blanked symbols represent storage modulus and loss modulus, respectively. (c) Digital photograph of the tensile testing specimens extruded at 160 °C (copolyamide and PSMS50) and 220 °C (PNS50).

Rheology measurements were performed to assess further the microstructural effects of OSA-*g*-starch in the copolyamide matrix and gain more insight into the processing characteristics of the developed biocomposites. First, the linear viscoelastic region was found through a strain sweep test conducted at 160 °C and a fixed angular frequency of 1 Hz. The data are plotted in Figure S6, where both plain matrix and biocomposites, except PSMS70, behaved independently of shear strain within the 0.01 to 100% strain range, indicating a linear viscoelastic region. Accordingly, a shear strain of 1% was selected for the frequency sweep test to guarantee that the measurement was in the linear viscoelastic regime. The frequency sweep results are illustrated in Figure 6a,6b, where the trend of storage (*G*′) and loss (*G*″) moduli, as well as complex viscosity (|η*|) were depicted as a function of angular frequency between 0.01 to 100 Hz at 160 °C. *G*″ dominated *G*′ at the test conditions, particularly at lower frequencies, indicating a fully relaxed state of the polymer chains, in which the molten polymers revealed more liquid-like or viscous behavior than solid-like or elastic ones,^91^ suggesting good processability of the compounds under the applied shear stress in the extruder.

The other parameter that reflects the processing flow performance of composite materials is complex viscosity.^92^ As shown in Figure 6b, a Newtonian plateau was observed in the low frequencies for all of the molten polymers, followed by a significant reduction at higher applied angular frequencies, specifying a non-Newtonian behavior known as shear-thinning viscosity, which could be due to the disentanglement of the molecular chains.^89,93^ This behavior suggests the excellent fluidity of the molten polymer in the extruder.^94^

Many researchers^11,38,89,91,92,95,96^ have reported a systematic increase in both complex viscosity and storage modulus of the matrix upon adding the filler due to the generation of a network-like structure inside the molten polymer, which led to the increase of the molecular entanglements of polymer chains, as well as owing to the interfacial adhesion and hydrogen bonding between the filler and polymer chains. Nevertheless, in this study, the complex viscosity and moduli decreased upon increasing OSA-*g*-starch content, indicating the lubricating effect of surface-treated starch. This trend could be attributed to the reduced entanglement of polyamide chains obtained by voluminous OSA molecules, which brought in more free volume into the polyamide matrix, as well as to the orientation of starch particles with flexible OSA chains along with the applied shear forces that promoted the chain mobilities, behaving thus similarly to previously reported for composites containing surface-modified lignin^93^ and clay^97^ particles.

To support the benefit of the surface treatment on the processability of the polyamide matrix, the viscosity of the composite containing 50 wt % native starch, i.e., PNS50, was included in Figure 6b. Contrastingly to modified starch, the addition of native starch increased the viscosity to at least 1 order of magnitude when compared to the composite with 50 wt % surface-modified starch, i.e., PSMS50. The increased viscosity made the PNS50 composite more challenging to process, and we applied a higher temperature (220 °C) to extrude this sample. As a result, this composite revealed significant discoloring compared to other biocomposites processed at 160 °C (Figure 6c), probably due to the partial decomposition of starch particles at such a high processing temperature. This observation firmly supports the benefit of the synthesized low-melting-point copolyamide for blending with biobased fillers, e.g., starch, as well as the advantage of the applied surface modification method in making starch particles compatible with polyamide matrix.

### Shape Memory Effect and Thermally Responsive Fabric Prototype

Shape memory polymers (SMPs) are a class of smart materials for which a temporary shape can be set, and the return to a permanent or partially recovered shape is further driven by exposure to an external stimulus, such as temperature change, light, voltage, and humidity.^98,99^ One-way shape memory polymers (1W-SMPs), as the name suggests, have the ability to return to their original or permanent shape from a fixed temporary shape in a one-time manner. Two-way shape memory polymers (2W-SMP), on the other hand, can actuate between two different shapes, typically a higher-temperature (’partially recovered’) shape and a low-temperature shape (temporary shape), upon exposure to the appropriate stimuli. The partially recovered shape is determined by geometry-defining netpoints in the polymer structure, which in the case of polyamide, is expected to be its crystalline domains.^100,101^ Similarly to 1W-SMPs, the polymer is initially deformed into a temporary shape at a temperature above its thermal resetting temperature, i.e., at the temperature that is above the melting point for geometry-defining domains. The polymer is then cooled under tension to lock it into its temporary shape, as in 1W-SMPs. To revert to its partially recovered shape, the 2W-SMP is heated to a transition temperature significantly below its thermosetting temperature, and cycling the temperature between low and transition temperatures will enable repeatable actuation.^99,102,103^

High activation temperature and low recovery are major drawbacks of many thermoplastic SMPs. For instance, Koerner et al.^104^ developed amorphous fluorinated polyimide with a high shape recovery temperature of 220 °C. Furthermore, Shi et al.^83^ synthesized *T*_g_-based SMP by introducing ionic moieties to polyether ether ketone (PEEK) with a switching temperature of around 200 °C. This work was expanded into a *T*_m_-based SMP by integrating sodium oleate, leading to a higher switching temperature of 230–240 °C resulting from the melting of sodium oleate. These limit their utilization in some applications, such as biomedicine, where fast recovery and low activation temperature close to the human body are essential.^101^ Additionally, from the point of view of shape memory textiles, many common textile materials cannot resist heating to the activation temperatures of the above-mentioned SMPs. Introducing plasticizers to the polymer matrix can affect the thermal properties of the SMPs, leading to a decrease in *T*_g_. For instance, plasticizers can weaken the H-bonds and reduce the transition temperature. Subsequently, the polymer chain movement at a specific temperature can increase.^105,106^ To this goal, in the current study, we developed a novel copolyoamide that presented a relatively low *T*_trans_ at around its *T*_g_, i.e., 55 °C. Furthermore, we incorporated surface-modified starch particles into the synthesized copolyamide not only to improve the green content of the synthesized polyamide but also to reduce the *T*_g_, which might lead to a decrease in the switching temperature of the polyamide.

**Figure 7:**
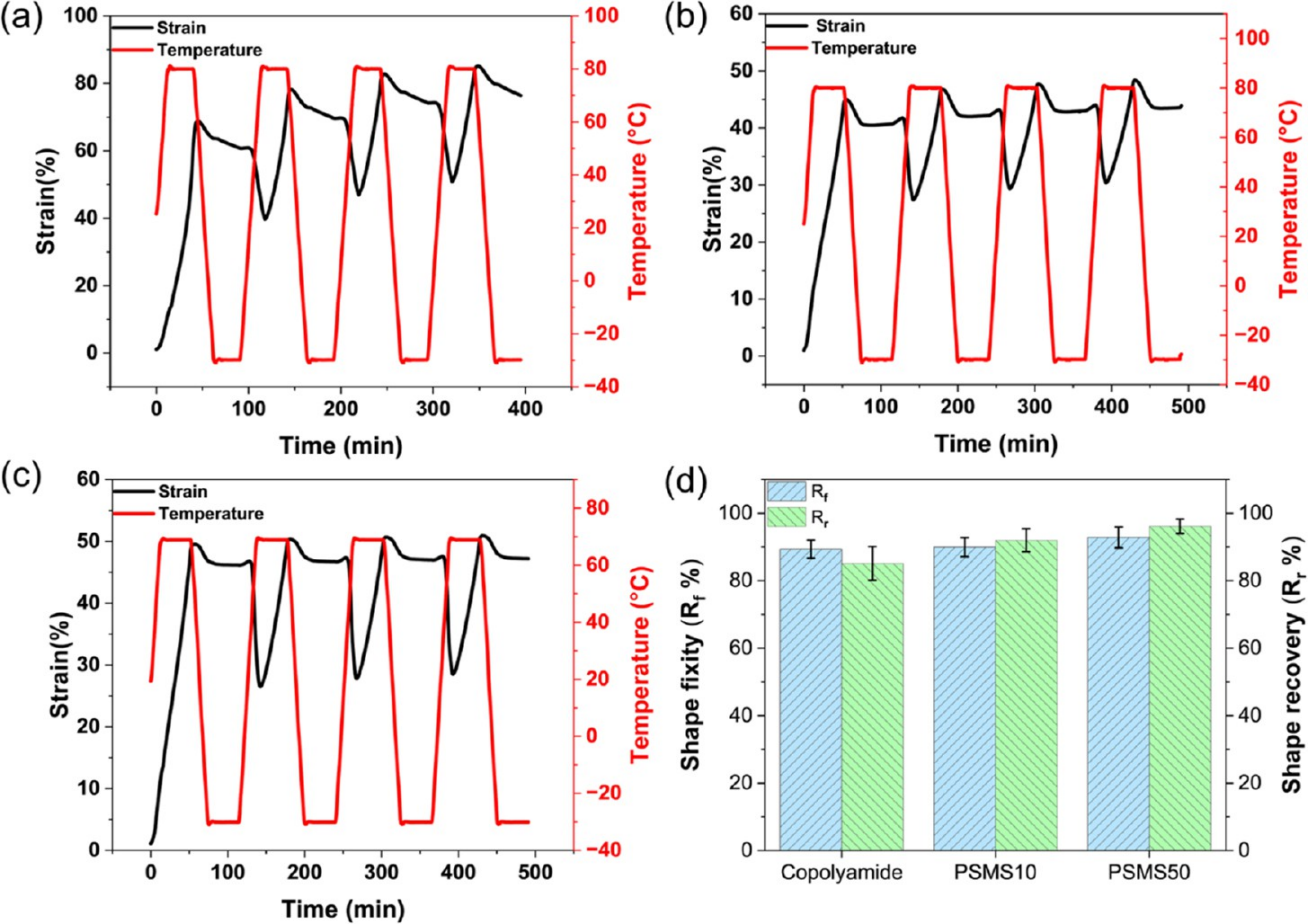
Strain/temperature versus time corresponds to the 4 shape memory cycles for (a) copolyamide, (b) PSMS10, and (c) PSMS50. (d) Shape recovery (*R*_r_) and shape fixity (*R*_f_) percentage for the plain copolyamide matrix and PSMS10 and PSMS50 biocomposites.

First, the 1W shape memory and shape recovery of the plain polymer and biocomposites, including 10 and 50% OSA-*g*-starch, were tested via a cyclic thermomechanical test. The recovery temperature was chosen to be *T*_g_ + 10 °C to allow for as complete recovery as possible. The results are provided in Figure 7 and Table S2. In the pure copolyamide matrix, *R*_r_ and *R*_f_ were, respectively, 85.13 ± 4.99 and 89.39 ± 2.68%, indicating excellent shape recovery properties of the newly synthesized copolyamide. The calculated values for both *R*_r_ and *R*_f_ were as high as reported for common shape memory polymers.^107,108^ High storage modulus below *T*_g_ and excellent rubber elasticity above *T*_g_ contributed to high *R*_r_ and *R*_f_. After 4 cycles, the film could still return to its original shape, indicating an excellent thermally induced shape memory.^109^

The effect of processing history could be seen in the first cycle; however, both *R*_r_ and *R*_f_ improved by increasing the number of test cycles, leading to the better shape memory effect resulting from memory stress reduction and shape memory training.^39,46^ During the first cycle, thermal shape recovery leads to a reduction in the entropy and disentanglement of some entanglements, diminishing the ability of polymer chains to return to their previous random coils. This stretching and loosening of physical entanglements result in fewer remaining entanglements available for disentanglement in subsequent cycles of thermomechanical loading. Consequently, the shape recovery value is lower during the first cycle compared to subsequent cycles.^110,111^

Both biocomposites, i.e., PSMS10 and PSMS50, revealed qualitatively similar behavior over heating/cooling cycles with relatively high 1W *R*_r_ and *R*_f_ values comparable with those of the unmodified copolyamide matrix. This confirms that the presence of starch particles did not interrupt polyamide chains’ mobility even at a relatively high filler content of 50 wt %, chiefly thanks to the employed compatibilization process. Both *R*_r_ and *R*_f_ were slightly enhanced in the biocomposites, demonstrating that the OSA-*g*-starch particles enhanced the mobility of the polymer chains and helped them easily arrange and recover to their original shape by disrupting the interchain supramolecular interactions. The degree of crystallinity (χ_c_) in shape memory polymers significantly influences their shape memory properties. Increased crystallinity content often results in a more pronounced and dependable shape memory effect.^112^ Consequently, the PSMS50 composite with relatively higher crystallinity exhibits slightly higher *R*_r_ and *R*_f_ values. However, it is essential to avoid excessive crystallinity, as it may render the polymer overly rigid, thus limiting its deformability and shape recovery capabilities. It is noteworthy that the shape recovery results for PSMS50 were obtained at a lower operational temperature, i.e., 70 °C. In other words, the incorporation of surface-modified starch particles not only improved the shape memory performance of the polyamide matrix but also significantly reduced the transition temperature, making the composite interesting for low-temperature actuating applications.

**Figure 8:**
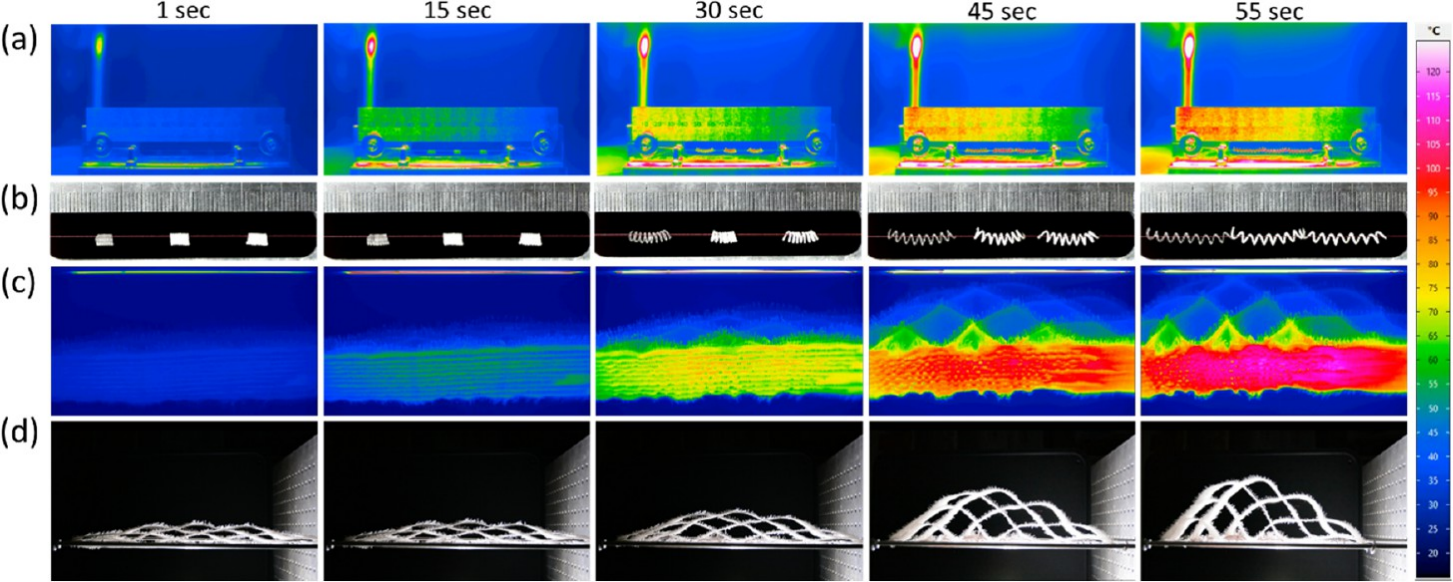
Actuation of heat-responsive fabric using PSMS50 yarn actuators exposed to an IR lamp. (a) IR and (b) digital images of PSMS50 yarn actuators. (c) IR and (d) digital images of heat-responsive fabric. The fabric undergoes five cycles of heating and cooling. The entire expansion and contraction process is provided in Video S1, Video S2, Video S3, and Video S4.

Owing to these biocomposites exhibited two distinct thermal transition temperatures, *T*_g_ and *T*_m_, the 2W shape memory effect was investigated. To showcase the potential application of the developed biocomposite in textiles, a heat-responsive fabric was created using thermos-responsive 2W shape memory PSMS50 actuators. The fabrication process involved the use of a custom-built twister-coiler device and thermosetting at a higher temperature of 120 °C, enabling the demonstration of repeatable thermal actuation. The 2W SM actuation of PSMS50 coils and the integrated heat-responsive fabric are depicted in Video S1, Video S2, Video S3, and Video S4. The choice of using coiled actuators is based on amplifying the thermally induced torsional motion of twisted filament when the filament is coiled, as explained previously by Haines et al.^5^ Additionally, Figure 8 illustrates their response to heating at specific time points.

Upon heating, the shape-morphing fabric exhibited rapid and significant heat-induced deformation, followed by successful recovery to its initial dimensions during the cooling process. Infrared thermal camera recordings, along with Video S3 and Video S4, showed that the shape-changing deformation initiated at approximately 50 °C, with the greatest range of movement achieved at around 100 °C. Notably, the fabric endured 5 heating/cooling cycles without any change in the actuation/recovery performance, confirming the durability of this smart textile.

The cotton yarns incorporated into the fabric provided the necessary flexibility to the top layer, facilitating contraction and expansion of the actuators. In essence, the cotton yarns, along with the plain weave structure, synergistically complemented and facilitated the actuation of the PSMS50 actuators within the textile network. This design allowed for the heat-driven alteration of a 3D textile and could be further utilized to reveal other hidden functionalities of functional yarns integrated into the nonactuated textile structure. Consequently, this construction concept enables the gradual exposure of new textile functionalities based on temperature variations.

In summary, the integration of the developed shape memory polymer actuators from the PSMS50 biocomposite into the textile structure showcased significant and heat-induced deformation. This approach provides a straightforward pathway for designing dynamic shape-changing textiles that enhance the capabilities of the current smart textiles.

## Conclusions

This study highlights the potential of utilizing biobased materials to address sustainability concerns associated with petroleum-based fibers in functional textile applications. A series of biocomposites consisting of surface-modified starch and polyamide were successfully developed. To prevent the thermal degradation of starch particles during the melt blending process, a novel low-melting-point copolyamide was synthesized by incorporating monomers with different alkyl chain lengths through copolymerization. The resulting copolyamide exhibited a significantly low melting point of 135 °C. To enhance the compatibility between starch particles and the copolyamide matrix, a simple, solvent-free method involving the grafting of octenyl succinic anhydride (OSA) onto the starch surface through esterification was employed. The successful grafting of the OSA was confirmed through various analyses, including FTIR, TGA, elemental analysis, and SEM images. Subsequently, different concentrations of OSA-*g*-starch, including a high concentration of 70 wt %, were successfully melt-blended with the copolyamide matrix at a relatively low processing temperature of 160 °C. The developed biocomposites were thoroughly characterized, and it was observed that the OSA-*g*-starch dispersed uniformly within the polymer matrix, resulting in improved interfacial adhesion and good compatibility. The biocomposites exhibited excellent stiffness/toughness balance, along with remarkable rheological properties and processability, even at a high filler loading of 70 wt %. Moreover, the shape memory and shape recovery properties of the pure polymer and biocomposites, including those with 10 and 50% OSA-*g*-starch, were evaluated through cyclic thermomechanical testing, confirming their outstanding shape recovery capabilities. Importantly, it was demonstrated that the shape memory behavior of the polyamide could be tailored and the actuation temperature could be reduced by increasing the starch particle content. In conclusion, this work presents the concept of developing responsive fabrics by integrating thermoresponsive shape memory polymers into textiles, thereby adding value to traditional textiles available in the market and paving the way for the advancement of smart and functional textiles.

## Data Availability

The data that support the findings of this study are available on request from the corresponding authors. The data are not publicly available due to privacy or ethical restrictions.

## Supporting Information Available

Starch elements calibration curve was obtained from elemental analysis results; TGA/derivative TG (TGA/DTG) thermograms of the starch and OSA-*g*-starch; GPC curves of the synthesized homopolymers and copolymer; DSC thermograms of the plain copolyamide and the composites with different concentrations of OSA-*g*-starch particles; strain sweep test was performed at a fixed angular frequency of 1 Hz at 160 °C; residue of the samples at 700 °C, extracted from TGA thermograms; and shape recovery (*R*_r_) and shape fixity (*R*_f_) of the samples at the second and third cycles (PDF)
Actuation of coils exposed to the infrared heat (recording was done by an FLIR SC660 thermal camera) (speed 25×) (MP4)
Actuation of coils exposed to the infrared heat (speed 25×) (MP4)
Actuation of the heat-responsive fabric exposed to the infrared heat (recording was done by an FLIR SC660 thermal camera) (speed 6×) (MP4)
Actuation of the heat-responsive fabric exposed to the infrared heat (speed 7×) (MP4)
