# Supplementary material for: Heat-Induced Actuator Fibers: Starch-Containing Biopolyamide Composites for Functional Textiles
Source: ACS Appl Mater Interfaces. 2023 Oct 3;15(41):48584–600. doi: 10.1021/acsami.3c08774 (PMC10591286; doi:10.1021/acsami.3c08774)
Supplement: Supplementary file 1 — am3c08774_si_001.pdf [file am3c08774_si_001.pdf]

Heat-Induced Actuator Fibers: Starch-Containing Bio-Polyamide Composites for Functional Textiles

Hossein Baniasadi[Ψ], *Zahra Madani*[Ψ], *Mithila Mohan, Maija Vaara, Sami Lipponen, Jaana Vapaavuori*[*], *Jukka V. Seppälä*[*]

Hossein Baniasadi - Polymer Technology, School of Chemical Engineering, Aalto University, Kemistintie 1, 02150, Espoo, Finland.

Zahra Madani - Department of Chemistry and Materials Science, School of Chemical Engineering, Aalto University, Kemistintie 1, 02150, Espoo, Finland.

Mithila Mohan - Department of Chemistry and Materials Science, School of Chemical Engineering, Aalto University, Kemistintie 1, 02150, Espoo, Finland.

Maija Vaara - Department of Chemistry and Materials Science, School of Chemical Engineering, Aalto University, Kemistintie 1, 02150, Espoo, Finland.

Sami Lipponen - Polymer Technology, School of Chemical Engineering, Aalto University, Kemistintie 1, 02150, Espoo, Finland.

Jaana Vapaavuori - Department of Chemistry and Materials Science, School of Chemical Engineering, Aalto University, Kemistintie 1, 02150, Espoo, Finland.

Jukka V. Seppälä - Polymer Technology, School of Chemical Engineering, Aalto University, Kemistintie 1, 02150, Espoo, Finland.

[*] Corresponding authors: jukka.seppala@aalto.fi and jaana.vapaavuori@aalto.fi

[Ψ] These authors have equal contributions.

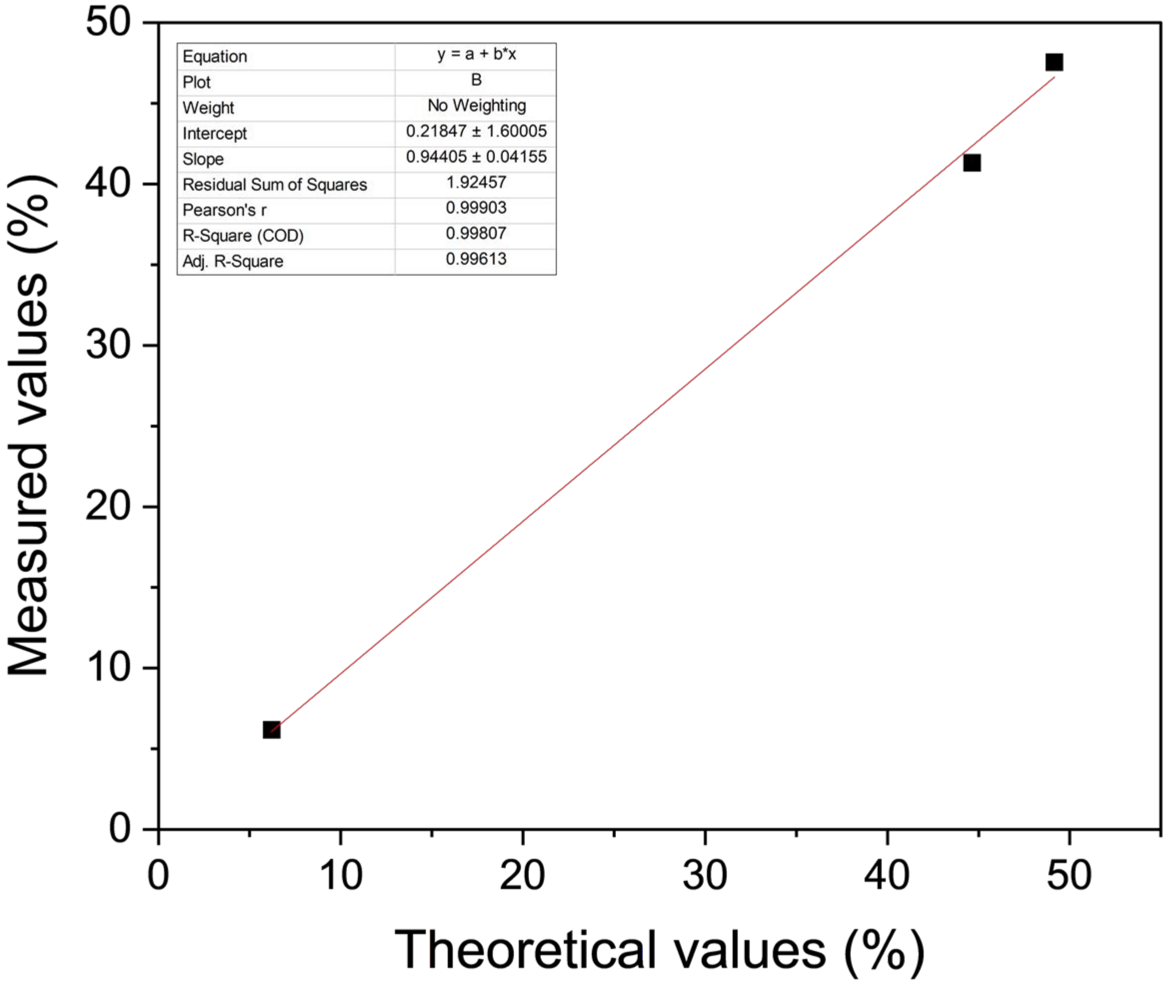

Figure S1. The starch elements calibration curve was obtained from elemental analysis results.

TGA/DTG of starch and OSA-*g*-starch

To monitor the thermal decomposition of starch before and after surface treatment, as a tool for qualitative evaluation of the grafting of OSA, TGA was conducted on starch before and after grafting (un-washed sample). The thermograms are plotted in **Figure S2**. The plain starch revealed two decomposition regions; a minor weight loss (~8%) below 120 ˚C corresponding to evaporation of physically and chemically bound water followed by a major decomposition (~80%) between 220 to 800 ˚C attributed to the pyrolytic decomposition phase of the biopolymers present (amylose and amylopectin), with an ash content of approximately 9%.[1–3] On the other side, OSA-*g*-starch presented three weight loss regions; a minor one (~ 2.7%)

below 120 ˚C, along with the second decomposition (~12%) between 200 to 280 ˚C, followed by a pyrolytic decomposition (~70%) within 280 to 800 ˚C, with an ash content of about 15%. Significant differences were observed in the TGA/DTG thermograms of the starch and OSA-*g*-starch. For instance, the weight loss below 200 ˚C, which corresponded to the evaporation of the trapped water, was considerably lower in the OSA-*g*-starch, indicating a more hydrophobic property of the surface-treated samples obtained by grafting of OSA molecules. Furthermore, the new stage appeared between 200 to 280 ˚C with the maximum decomposition temperature at ~260 ˚C due to the decomposition of the grafted OSA molecules.[4] This decomposition temperature indicated that the surface-modified starch was amenable to the newly developed low melting-point copolyamide. Moreover, the ash content in the surface-treated sample was higher, which could be explained by the fact that the residue in the OSA-*g*-starch was a mixture of more ordered crystallites that required higher thermal temperatures for decomposition.[5]

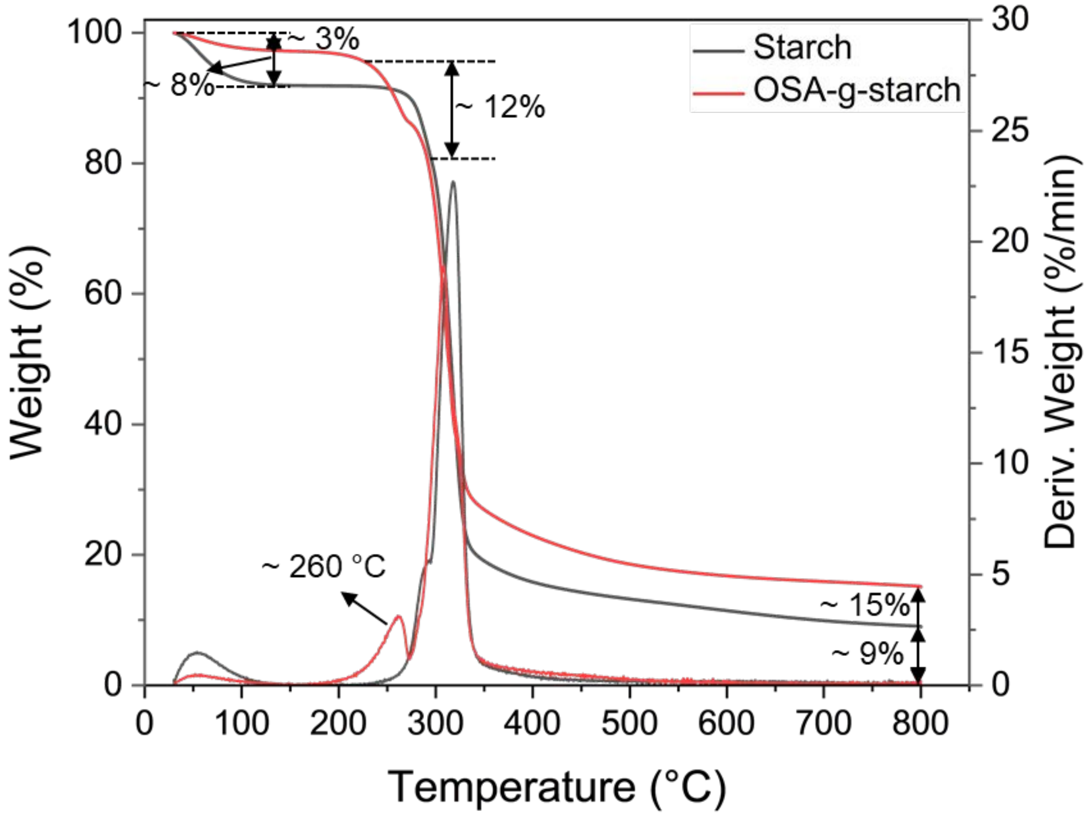

Figure S2. TGA/DTG thermograms of the starch and OSA-*g*-starch.

TGA/DTG of the matrix and biocomposites

As depicted in **Figure S3**, the copolyamide presented a typical polyamide decomposition profile with less than 1% residue at 700 ˚C.[6] Although it was thermally stable below 400 ˚C, a rapid weight loss occurred between 400 to 500 ˚C owing to the decomposition of the polymeric backbone with a maximum decomposition at 460 ˚C. For block copolymers with different polymer blocks, double DTG peaks have been reported in the literature; nevertheless, a unimodal decomposition profile observed in the current study could suggest an alternating or random copolymer formation.[7] It is worth notifying that the absence of any other decomposition peak in the copolyamide DTG curve could support the fact that no oligomers/polymers rather than polyamide were synthesized during the copolymerization reaction. On the other side, the biocomposites revealed two pronounced decomposition stages. The first one took shape between 300 to 400 ˚C with a maximum decomposition temperature of ~304 ˚C and the second one appeared between 400 to 500 ˚C with a maximum

decomposition of ~460 ˚C. In other words, the biocomposites presented the decomposition stages of both OSA-*g*-starch and copolyamide. For instance, PSMS30 presented approximately 22% weight loss before 400 ˚C due to the starch decomposition, along with about 75% weight loss between 400 to 500 ˚C, attributed to the polyamide degradation (**Figure S3c**). Obviously, the height of the first DTG peak increased upon increasing the bio-filler content, while the second one reduced, indicating an increase in the starch portion. Finally, the first decomposition stage prevailed in the biocomposite with a higher starch content, i.e., 70 wt.%. Besides, the residue material (**Table S2**) increased with the increase in the starch content. Concerning the residue of the copolyamide and OSA-*g*-starch at 700 ˚C, the residue of biocomposites had good agreement with the experimental concentration of OSA-*g*-starch, suggesting the effectiveness of the applied melt blending process in distributing the particles into the polymer matrix evenly.

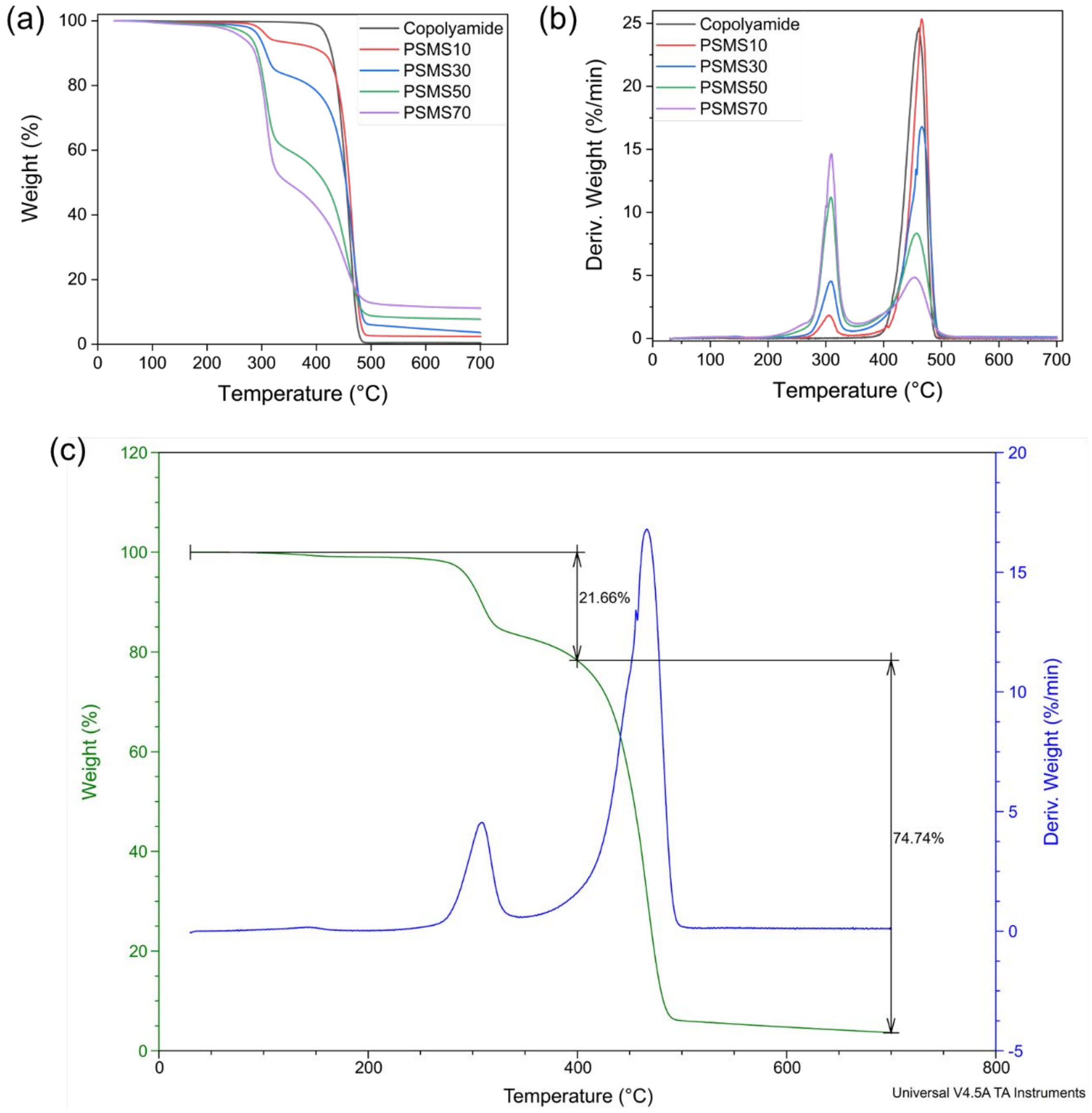

Figure S3. a) TGA, b) DTG thermograms of the synthesized copolyamide and biocomposites, and c) TGA/DTG curves of the PSMS30.

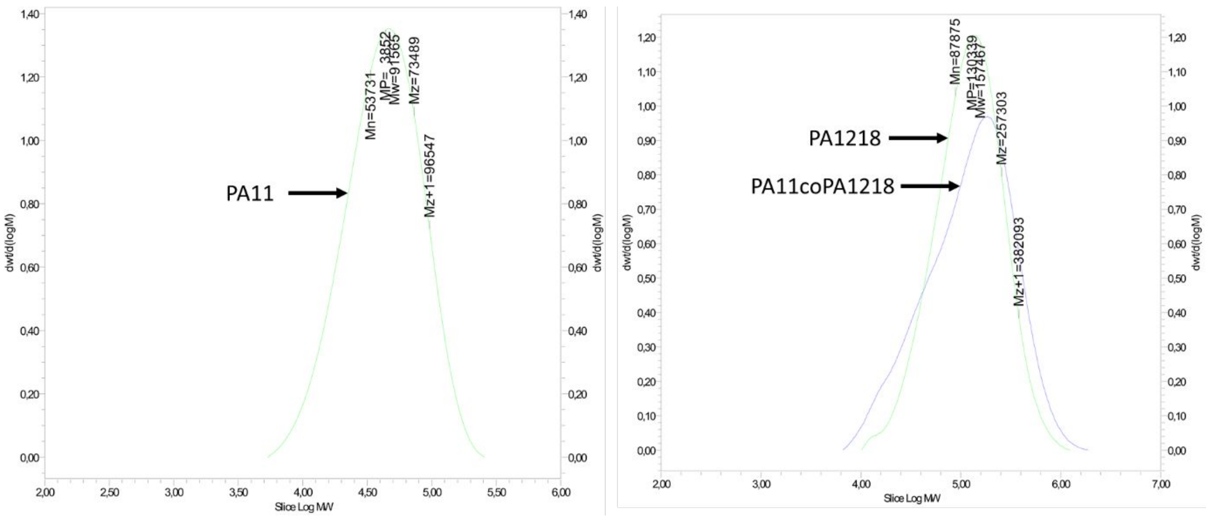

Figure S4. GPC curves of the synthesized homopolymers and copolymer.

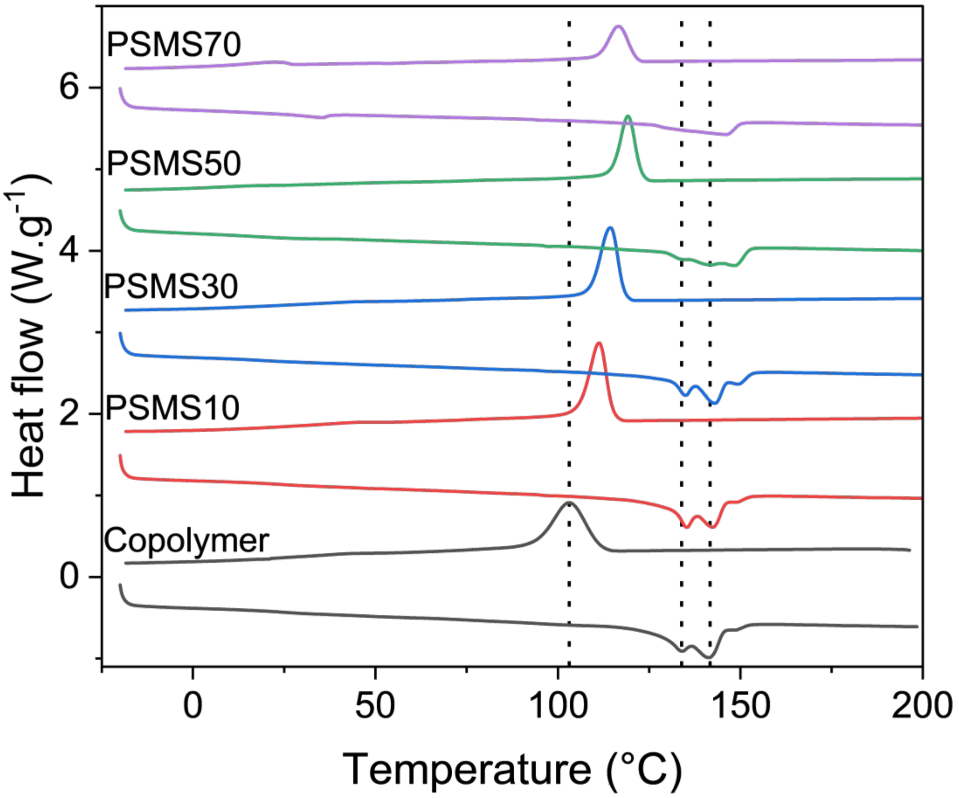

Figure S5. The DSC thermograms of the plain copolyamide and the composites with different concentrations of OSA-g-starch particles.

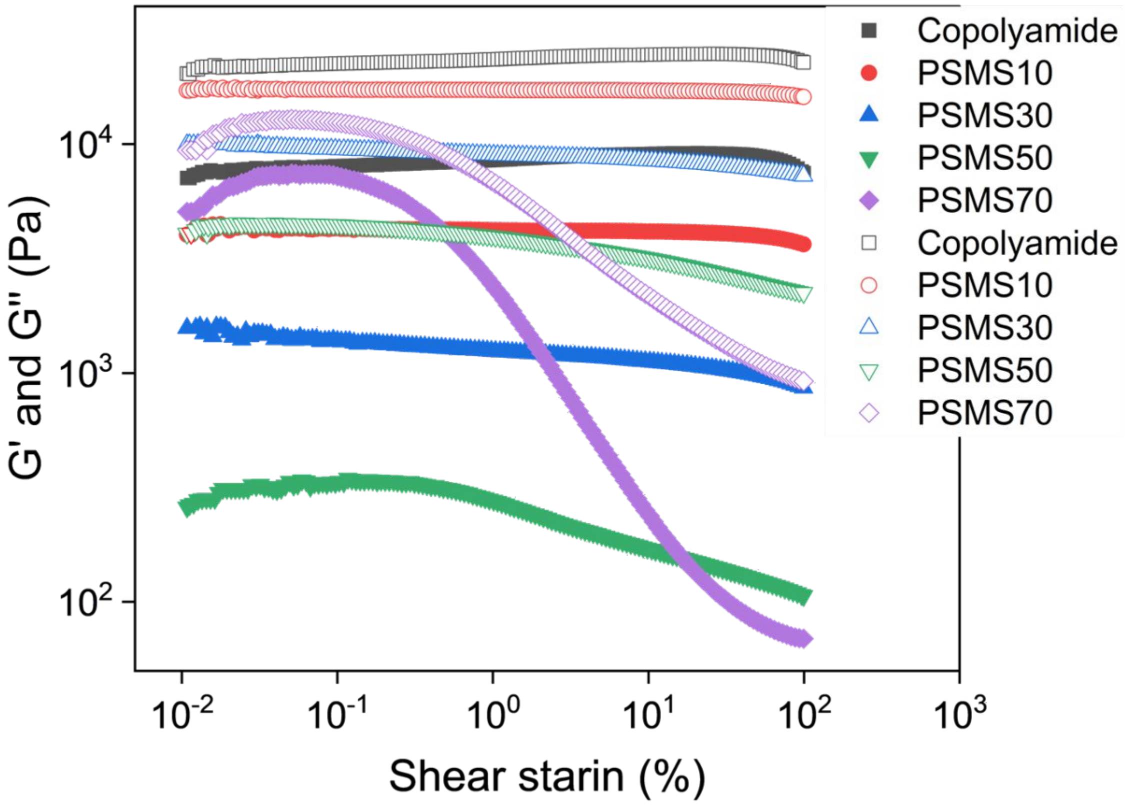

Figure S6. The strain sweep test was performed at a fixed angular frequency of 1 Hz at 160 ˚C.

Table S1. The residue of the samples at 700 ˚C, extracted from TGA thermograms

Sample	Residue[I] at 700 ˚C (%)	Residue[II] at 700 ˚C (%)
Starch	9.5	---
OSA-g-starch	9.3	---
Copolyamide	0.5	---
PSMS10	2.5	1.9
PSMS30	3.5	4.8
PSMS50	7.8	7.7
PSMS70	11.2	10.6

[I] Extracted from TGA thermograms.

[II] Calculated due to the portion of each component and their residue at 700 ˚C.

Table S2. Shape recovery (R_r) and shape fixity (R_f) of the samples at the second and third cycles.

Sample	R_{r2}(%)	R_{r3}(%)	R_{f2}(%)	R_{f3}(%)
Copolymer	81.59	88.66	89.20	89.58
PSMS10	89.56	94.38	90.14	89.74
PSMS50	94.61	97.62	93.05	92.61

Video S1. Actuation of coils exposed to the infrared heat (recording was done by thermal camera FLIR SC660 thermal Camera) (speed 25X).

Video S2. Actuation of coils exposed to the infrared heat (speed 25X).

Video S3. Actuation of the heat-responsive fabric exposed to the infrared heat (recording was done by thermal camera FLIR SC660 thermal Camera) (speed 6X).

Video S4. Actuation of the heat-responsive fabric exposed to the infrared heat (speed 7X).

References

(1) Ruggero, F.; Carretti, E.; Gori, R.; Lotti, T.; Lubello, C. Monitoring of Degradation of Starch-Based Biopolymer Film under Different Composting Conditions, Using TGA, FTIR and SEM Analysis. *Chemosphere* **2020**, *246*, 125770.

(2) Morales, P. A. M.; Rodríguez, Á. M. M.; Pardo, L. N. M.; Vargas, B.; Osorio, B. L. L. Cassava and Banana Starch Modified with Maleic Anhydride-Poly (Ethylene Glycol) Methyl Ether (Ma-MPEG): A Comparative Study of Their Physicochemical Properties as Coatings. *Int. J. Biol. Macromol.* **2022**.

(3) Sukkaneewat, B.; Panrot, T.; Rojruthai, P.; Wongpreedee, T.; Prapruddivongs, C. Plasticizing Effects from Citric Acid/Palm Oil Combinations for Sorbitol-Crosslinked Starch Foams. *Mater. Chem. Phys.* **2022**, 125732.

(4) Don, T.-M.; Li, T.-S.; Lai, W.-C. Miscibility and Flexibility of Poly (Lactic Acid) Blends with Octadecenylsuccinic Anhydride. *Polym. Degrad. Stab.* **2019**, *162*, 55–65.

(5) Mapengo, C. R.; Ray, S. S.; Emmambux, M. N. Granular Morphology, Molecular Structure and Thermal Stability of Infrared Heat-Moisture Treated Maize Starch with Added Lipids. *Food Chem.* **2022**, *382*, 132342.

(6) Mao, G.; Liu, T.; Chen, Y.; Gao, X.; Qin, J.; Zhou, H.; Jin, W. Polyamide@ GO Microporous Membrane with Enhanced Permeability for the Molecular Sieving of Nitrogen over VOC. *J. Memb. Sci.* **2022**, 120443.

(7) PÚrez, A.; Lligadas, G.; Ronda, J. C.; GaliÓ, M.; Cßdiz, V. Thermoreversible Poly (Trimethylene Carbonate)-Based Block Copolymers Containing Reactive Furfuryl Groups via Sequential ROP and ATRP. *Eur. Polym. J.* **2022**, *162*, 110873.
